# A dynamic spike threshold with correlated noise predicts observed patterns of negative interval correlations in neuronal spike trains

**DOI:** 10.1007/s00422-022-00946-5

**Published:** 2022-10-16

**Authors:** Robin S. Sidhu, Erik C. Johnson, Douglas L. Jones, Rama Ratnam

**Affiliations:** 1 Department of Electrical and Computer Engineering, University of Illinois at Urbana-Champaign, Urbana, IL USA; 2 The Johns Hopkins University Applied Physics Laboratory, Laurel, MD USA; 3 Division of Biological and Life Sciences, School of Arts and Sciences, Ahmedabad University, Ahmedabad, Gujarat India

**Keywords:** Negative interspike interval correlations, Serial correlation coefficients, Dynamic threshold, Noisy threshold, Adaptation, Optimal neural coding, P-type electrosensory afferents, Weakly electric fish

## Abstract

Negative correlations in the sequential evolution of interspike intervals (ISIs) are a signature of memory in neuronal spike-trains. They provide coding benefits including firing-rate stabilization, improved detectability of weak sensory signals, and enhanced transmission of information by improving signal-to-noise ratio. Primary electrosensory afferent spike-trains in weakly electric fish fall into two categories based on the pattern of ISI correlations: non-bursting units have negative correlations which remain negative but decay to zero with increasing lags (Type I ISI correlations), and bursting units have oscillatory (alternating sign) correlation which damp to zero with increasing lags (Type II ISI correlations). Here, we predict and match observed ISI correlations in these afferents using a stochastic dynamic threshold model. We determine the ISI correlation function as a function of an arbitrary discrete noise correlation function ${{\,\mathrm{\mathbf {R}}\,}}_k$, where *k* is a multiple of the mean ISI. The function permits forward and inverse calculations of the correlation function. Both types of correlation functions can be generated by adding colored noise to the spike threshold with Type I correlations generated with slow noise and Type II correlations generated with fast noise. A first-order autoregressive (AR) process with a single parameter is sufficient to predict and accurately match both types of afferent ISI correlation functions, with the type being determined by the sign of the AR parameter. The predicted and experimentally observed correlations are in geometric progression. The theory predicts that the limiting sum of ISI correlations is $-0.5$ yielding a perfect DC-block in the power spectrum of the spike train. Observed ISI correlations from afferents have a limiting sum that is slightly larger at $-0.475 \pm 0.04$ ($\text {mean} \pm \text {s.d.}$). We conclude that the underlying process for generating ISIs may be a simple combination of low-order AR and moving average processes and discuss the results from the perspective of optimal coding.

## Introduction

The spiking activity of many neurons exhibits memory, which stabilizes the neurons’ firing rate and makes it less variable than a renewal process. In spontaneously active neurons, a signature of these memory effects can be found in the serial correlations of interspike intervals (ISIs), which display a prominent negative correlation between adjacent ISIs. This is a result of long intervals following short intervals so that fluctuations from the mean ISI are damped over long time-scales, thereby stabilizing the firing rate (Ratnam and Nelson 2000; Goense and Ratnam 2003). Negative correlation between adjacent ISIs, which is the first serial correlation coefficient ($\rho _1$), can assume a range of values (Farkhooi et al. 2009) from near-zero [close to a renewal spike train, e.g., Lowen and Teich (1992); Fisch et al. (2012)] to values close to $-0.9$ (Ratnam and Nelson 2000). While more negative values may suggest a stronger memory effect, the relationship between the extent of memory in the spike train and their ISI correlations is by no means clear, in part due to the difficulty in determining joint interval distributions of arbitrarily high orders (van der Heyden et al. 1998; Ratnam and Nelson 2000).

Negative correlations in spontaneous spike trains, or in spike trains obtained under quiescent conditions, have been known for many years. Early reports came from lateral line units of Japanese eel (Katsuki et al. 1950), the retina of the cat (Kuffler et al. 1957), and subsequently from several neuronal systems (Farkhooi et al. 2009; Neiman and Russell 2001; Johnson et al. 1986; Lowen and Teich 1992; Hagiwara and Morita 1963; Amassian et al. 1964; Bullock and Chichibu 1965; Calvin and Stevens 1968; Nawrot et al. 2007; Fisch et al. 2012) (see Avila-Akerberg and Chacron 2011; Farkhooi et al. 2009, for tabulations of negative correlations). In the active sense of some wave-type weakly electric fish (Hagiwara and Morita 1963; Bullock and Chichibu 1965), primary electrosensory afferents exhibit the strongest known correlations in their adjacent ISIs (Ratnam and Nelson 2000). These electrosensory neurons are an excellent model system for studying memory effects and regularity in firing due to their high spontaneous spike-rates (Chacron et al. 2001; Ratnam and Nelson 2000; van der Heyden et al. 1998). It is likely that spike encoders which demonstrate negative ISI correlations have adaptive value because they can facilitate optimal detection of weak sensory signals (Goense and Ratnam 2003; Goense et al. 2003; Ratnam and Nelson 2000; Ratnam and Goense 2004; Chacron et al. 2001; Hagiwara and Morita 1963; Nesse et al. 2010; Farkhooi et al. 2011) and enhance information transmission (Chacron et al. 2005, 2004b, a).

Negative ISI correlations are a characteristic feature of spike frequency adaptation where a constant DC-valued input to a neuron is gradually forgotten, possibly due to an intrinsic high-pass filtering mechanism in the encoder (Liu and Wang 2001; Benda and Herz 2003; Benda et al. 2010). Two commonly used models of spike frequency adaptation are based on: (1) a dynamic threshold, and (2) an adaptation current, both of which cause increased refractoriness following an action potential. In the case of a dynamic threshold, the increased refractoriness is due to an elevation in firing threshold, historically referred to as “accommodation” (Hill 1936). This is usually modeled as an abrupt increase in spike threshold immediately after a spike is output, followed by a relaxation of the threshold to its normative value without reset (Buller 1965; Hagiwara 1954; Goldberg et al. 1964; Geisler and Goldberg 1966; Ten Hoopen 1964; Brandman and Nelson 2002; Chacron et al. 2000, 2001; Schwalger et al. 2010; Schwalger and Lindner 2013). Thus, if two spikes are output in quick succession with an interval smaller than the mean ISI, the upward threshold shift will be cumulative, making the membrane more refractory, and so a third spike in the sequence will occur with an ISI that is likely to be longer than the mean. In this way, a dynamic time-varying threshold serves as a moving barrier, carrying with it a memory of prior spiking activity. In the case of an adaptation current, outward potassium currents, including voltage gated potassium currents and calcium-dependent or AHP currents, can give rise to increased refractoriness and lead to spike frequency adaptation (e.g., Benda and Herz 2003; Prescott and Sejnowski 2008; Liu and Wang 2001; Benda et al. 2010; Jolivet et al. 2004; Schwalger et al. 2010; Fisch et al. 2012). Adaptation currents are usually modeled by explicitly introducing a hyperpolarizing outward current with a time-varying conductance in the equation for the membrane potential. The conductance (or a gating variable) will be elevated following a spike and, as with a dynamic threshold, it will decay back to its normative value. Both models have been successful in reproducing nearly identical spike frequency adaptation behavior for a step input current, and normalized first ISI correlation coefficient ($\rho _1$), although they differ in other details (Benda et al. 2010).

In this report, we focus on a dynamic threshold model. A simple dynamic threshold model can accurately predict spike-times in cortical neurons (Kobayashi et al. 2009; Gerstner and Naud 2009; Jones et al. 2015) and peripheral sensory neurons (Jones et al. 2015). Further, we had recently proposed that spike trains encoded with a dynamic threshold are optimal, in the sense that they provide an optimal estimate of the input by minimizing coding error (estimation error) for a fixed long-term spike-rate (energy consumption) (Jones et al. 2015; Johnson et al. 2015, 2016). These results did not incorporate noise in the model, and so here, we extend our earlier model by incorporating noise to model spike timing variability and serial ISI correlations.

In previous work, colored noise or Gaussian noise (or both) is added to a dynamic threshold, or to an adaptation current, or to the input signal so that negative correlations can be observed (Brandman and Nelson 2002; Chacron et al. 2001, 2003; Prescott and Sejnowski 2008). In these models the first ISI correlation coefficient $\rho _1$ (between adjacent ISIs) is close to or equal to $-0.5$, and all remaining correlation coefficients $\rho _i$, $i \ge 2$, are identically zero. In another report (Benda et al. 2010) $\rho _1$ is parameterized, and can assume values between 0 and $-0.5$. Experimental spike trains demonstrate broader trends, where $\rho _1$ can assume values smaller or greater than $-0.5$, and the remaining coefficients can be nonzero for several lags, sometimes with damped oscillations and sometimes monotonically increasing to zero (Ratnam and Nelson 2000). In several types of integrate-and-fire models with adaptation currents (Schwalger et al. 2010; Schwalger and Lindner 2013), colored and Gaussian noise fluctuations of different time-scales (slow and fast, respectively) determine the various patterns of ISI correlations, including positive correlations. All of these patterns had a geometric structure (i.e., $\rho _k/\rho _{k-1} = \text {constant}$). Urdapilleta (2011) also obtained a geometric structure with monotonically decaying correlation pattern with $\rho _k < 0$. These latter studies show that the role of noise fluctuations, in particular the time-scale of fluctuations, is important in determining patterns of ISI correlations. So, far adaptation models with an adaptation current have successfully predicted patterns of ISIs, but models with dynamic thresholds have been investigated much less, and it is not known whether they can accurately predict observed ISI patterns. Models that can accurately predict experimentally observed correlation coefficients for all lags have the potential to isolate mechanisms responsible for ISI fluctuations and negative correlations and provide insights into neural coding. This is the goal of the current work.

Serial interspike interval correlations observed in primary P-type afferents of weakly electric fish *Apteronotus leptorhynchus* are modeled using a dynamic threshold with noise. The model is analytically tractable and permits an explicit closed-form expression for ISI correlations in terms of an arbitrary correlation function ${{\,\mathrm{\mathbf {R}}\,}}_k$, where *k* is a multiple of the mean ISI. This allows us to solve the inverse problem where we can determine ${{\,\mathrm{\mathbf {R}}\,}}_k$ given a sequence of observed correlation coefficients. Theoretically, the limiting sum of ISI correlation coefficients is $-0.5$ (a perfect DC-block), and experimental correlation coefficients are close to this sum. This model is parsimonious, and in addition to predicting spike-times as shown earlier, it reproduces observed ISI correlations. Finally, the model provides a fast method for generating surrogate spike trains that match a mean firing rate with prescribed ISI distribution, joint-ISI distribution, and ISI correlations.

## Methods

### Experimental procedures

Spike data from P-type primary electrosensory afferents were collected in a previous *in vivo* study in *Apteronotus leptorhynchus*, a wave-type weakly electric fish (Ratnam and Nelson 2000). All animal procedures including animal handling, anesthesia, surgery, and euthanasia received institutional ethics (IACUC) approval from the University of Illinois at Urbana-Champaign and were carried out as previously reported (Ratnam and Nelson 2000). No new animal procedures were performed during the course of this work. Of relevance here are some details on the electrophysiological recordings. Briefly, action potentials (spikes) were isolated quasi-intracellularly from P-type afferent nerve fibers in quiescent conditions under ongoing electric organ discharge (EOD) activity (this is the so-called baseline spiking activity of P-type units). An artificial threshold was applied to determine spike onset times, and reported at the ADC sampling rate of $16.67~\text {kHz}$ (sampling period of $60~\mu \text {s}$). The fish’s ongoing quasi-sinusoidal EOD was captured whenever possible to determine the EOD frequency from the power spectrum, or the EOD frequency was estimated from the power spectrum of the baseline spike train. Both methods reported almost identical EOD frequencies. EOD frequencies typically ranged from 750 to 1000 Hz (see Ratnam and Nelson 2000, for more details).

### Data analysis

P-type units fire at most once every EOD cycle, and this forms a convenient time-base to resample the spike train (Ratnam and Nelson 2000). Spike times resampled at the EOD rate are reported as increasing integers. Resampling removes the phase jitter in spike-timing but retains long-term correlations due to memory effects in the spike train. Spike-times were converted to a sequence of interspike intervals, $X_1,\,X_2,\,\ldots ,\,X_k,\ldots , X_N$.

#### Autocorrelation function

The normalized autocorrelation function for the sequence of ISIs are the normalized serial correlation coefficients or SCCs, $\rho _1,\,\rho _2,\,\rho _3,\,\ldots$, with $\rho _0 = 1$ by definition. These were estimated from time-stamps at the original ADC rate of $16.67~\text {kHz}$ and at the resampled EOD frequency (Ratnam and Nelson 2000). In some afferents, there is a small difference between the two estimates, particularly in the estimates of $\rho _1$, but this has negligible effect on the results. The normalized ISI correlation function (i.e., correlation coefficients) were estimated from the resampled spike trains as follows:

1 $$\begin{aligned} \rho _k = \frac{\sum _{i=1}^{M-k}\,(X_i - T_1)(X_{i+k} - T_1)}{\left( \sum _{i=1}^{M-k}\,(X_i - T_1)^2\, \sum _{i=1}^{M-k}\,(X_{i+k} - T_1)^2\right) ^{1/2}}, \quad k \ge 0, \end{aligned}$$

where $T_1$ is the mean ISI, and *M* is the number of spikes in a block, typically ranging from 1000–3000 spikes. Correlation coefficients were estimated in non-overlapping blocks and then averaged over [*N*/*M*] blocks. There was no drift in the mean ISI within a block. We usually had about $2.5 \times 10^5$ spikes per afferent.

Throughout this work, we will use the term covariance to refer to the mean subtracted cross-correlation. If the two random variables in question are identical, then we will simply refer to covariance as variance, and correlation as autocorrelation. If the correlation is normalized by the variance, then we will refer to it as the correlation coefficient. The abbreviation SCCs stand for ISI serial correlation coefficients and are the same as the normalized ISI autocorrelation function (ACF).

#### Partial autocorrelation function

In addition to the normalized autocorrelation (SCCs), we compute the normalized partial autocorrelation $\phi _{k,\,k}$ between $X_i$ and $X_{i+k}$ by removing the linear influence of the intervening variables $X_{i+1},\,\ldots ,\,X_{i+k-1}$ (Box and Jenkins 1970). The notation $\phi _{k,\,j}$ means that the process is purely autoregressive (AR) of order *k*, and $\phi _{k,\,j}$ is the *j*th coefficient in the AR model. Partial autocorrelations provide a convenient way to identify an AR process, just as the autocorrelation function, Eq. (1), provides a way to identify a moving average (MA) process. When the ISIs are AR of order-*p*, then the partial correlation function (PACF) is finite with $\phi _{k,\,k} = 0$, for $k > p$, however, the autocorrelation function will be infinite. Conversely, when the process is MA of order-*m*, the autocorrelation function is finite with $\rho _k = 0$, for $k > m$, however, the partial autocorrelation function will be infinite. When the partial autocorrelation and autocorrelation functions are both infinite, the underlying process is neither purely AR nor purely MA, but is an autoregressive moving average (ARMA) process of some unknown order $(p,\,m)$. The partial autocorrelations $\phi _{1,\,1},\,\phi _{2,\,2},\,\phi _{3,\,3},\,\ldots$, can be obtained for $k = 1,\,2,\,3,\,\ldots$, by solving the Yule-Walker equations. A more efficient method is to solve Durbin’s recursive equations. Durbin’s formula is (Box and Jenkins 1970),

2 $$\begin{aligned} \phi _{k+1,\,j}= & {} \phi _{k,\,j} - \phi _{k+1,\,k+1}\,\phi _{k,\,k+1-j}, \quad j = 1,\,2,\,\ldots ,\,k, \end{aligned}$$

3 $$\begin{aligned} \phi _{k+1,\,k+1}= & {} \frac{\rho _{k+1} - \sum _{j=1}^{k}\,\phi _{k,\,j}\,\rho _{k+1-j}}{1 - \sum _{j=1}^{k}\,\phi _{k,\,j}\rho _j}, \end{aligned}$$

4 $$\begin{aligned} \phi _{1,\,1}= & {} \rho _1, \end{aligned}$$

where the $\rho _j$ are SCCs obtained from Eq. (1). The $\phi _{k,\,j}$ in the formula are estimates with some mean and standard deviation over the population. To reduce the estimation error, we can follow the same procedure as for serial correlations by averaging over blocks.

**Fig. 1:**
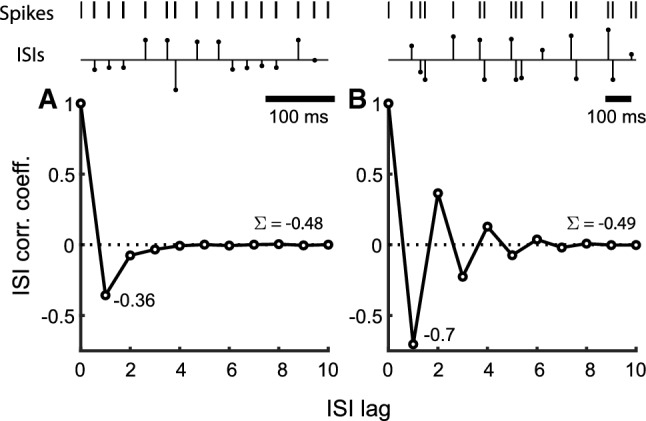
Representative normalized interspike interval (ISI) correlation functions from two P-type primary electrosensory afferent spike trains from two fish. For each column, from top to bottom, panels depict a sample stretch of spikes, sequence of ISIs for the sample spikes (normalized to mean ISI), and the pattern of ISI correlations. **A** Type I ($\rho _1 > -0.5$): non-bursting unit with first ISI correlation coefficient $\rho _1 = -0.36$. Remaining $\rho _k < 0$ diminish to 0. Sum of correlation coefficients ($\Sigma$) over 15 lags is $-0.48$. **B** Type II ($\rho _1 < -0.5$): Strongly bursting unit with $\rho _1 = -0.7$ with marked alternating positive and negative correlations. Sum of correlation coefficients ($\Sigma$) over 15 lags is $-0.49$. Spike trains sampled at $60~\mu \text {s}$. Mean ISI ± SD (in ms): $2.42 \pm 0.72$ (**A**), and $6.04 \pm 3.59$ (**B**). Electric organ discharge (EOD) frequency: 948 Hz (**A**), and 990 Hz (**B**)

**Fig. 2:**
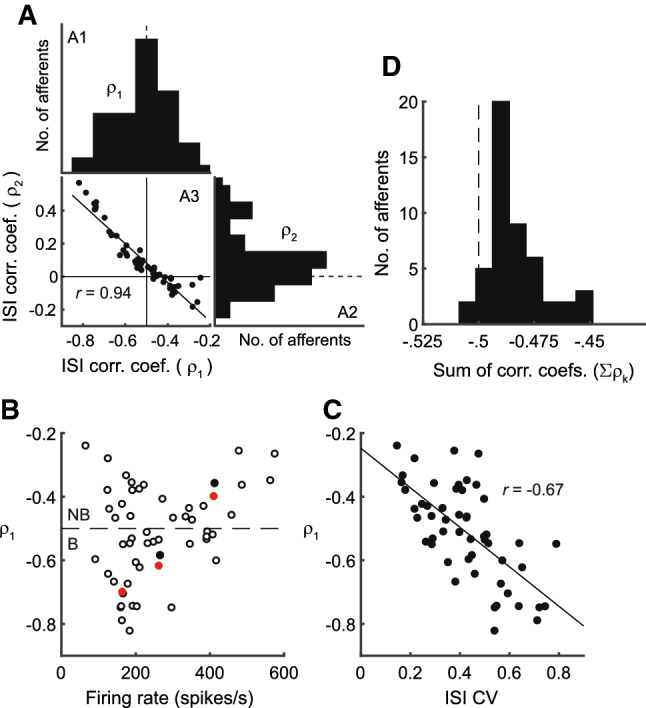
Population summaries of normalized ISI correlation function for P-type primary afferent spike-trains ($N = 52$). **A**. Summaries of first and second ISI correlation coefficients. A1 Histogram of correlation coefficient between adjacent ISIs ($\rho _1$), $\bar{\rho }_1 = -0.52 \pm 0.14$ ($\text {mean} \pm \text {s.d.}$), range $-0.82$ to $-0.25$ A2. Histogram of correlation coefficient (rotated counter clockwise by $90^{\text {o}}$) between every other ISI ($\rho _2$), $\bar{\rho }_2 = 0.10 \pm 0.18$, range $-0.18$ to 0.57. A3. Anti-diagonal relationship between observed $\rho _1$ (abscissa) and $\rho _2$ (ordinate) (filled circles). Line describes best fit, $\rho _2 = -1.18\rho _1 -0.51$ with $\text {Pearson's } r = 0.94$. **B** $\rho _1$ as a function of firing rate. Filled black circles are the three exemplar neurons considered in this work, and the filled red circles are their matched models. **C** $\rho _1$ as a function of coefficient of variation (CV) of ISIs with $\text {Pearson's } r = -0.67$. **D** Mean sum of correlation coefficients for the population over 15 lags, $\sum \,{\rho _k} = -0.475 \pm 0.04$. The population histograms in panels A1 and A2 were reported earlier with a different bin width (see Ratnam and Nelson 2000, Figs. 7A and B therein, respectively)

## Results

### Experimental results

ISI serial correlation coefficients (SCCs) were estimated from the baseline activity of 52 P-type primary electrosensory afferents in the weakly electric fish *Apteronotus leptorhynchus* (see Materials and Methods). SCCs from two example afferents (Fig. 1) demonstrate the patterns of negative SCCs observed in spike trains, and motivate this work. Statistical properties of these and other spike trains were reported earlier (Ratnam and Nelson 2000), with a qualitative description of SCCs and some descriptive statistics. A complete analytical treatment is undertaken here. Two types of serial interspike interval statistics can be identified according to the value taken by $\rho _1$ (the first SCC, between adjacent ISIs).

1. Type I: $-0.5< \rho _1 < 0$. Subsequent $\rho _k$ are negative and diminish to 0 with increasing *k* (Fig. 1A). The ISIs of these afferents are unimodal (shown later) and their spike trains do not exhibit bursting activity.
2. Type II: $\rho _1 < -0.5$. Subsequent $\rho _k$ alternate between positive ($\rho _{2k})$ and negative ($\rho _{2k+1}$) values, are progressively damped, and diminish to zero (Fig. 1C). The ISIs of these spike trains are bimodal with a prominent peak at an ISI equal to about one period of the electric organ discharge (EOD) (shown later). These spike trains exhibit strong bursting.

Afferent fibers sampled from individual fish were a mix of Types I and II. Additionally we identify a third type that has not been observed in experimental data (at least by these authors) but is fairly common in some models with adaptation (e.g., Brandman and Nelson 2002; Chacron et al. 2001, see also Discussion). We call this Type III, and for this pattern of SCCs $\rho _1 = -0.5$ and subsequent $\rho _k$ are identically zero (Fig. 1). The Type III pattern is a singleton (i.e., there is only one SCC pattern in this class).

The baseline spike-train statistics of 52 P-type afferents reported earlier (Ratnam and Nelson 2000) were analyzed in detail in this work. All observed units showed a negative correlation between succeeding ISIs ($\rho _1 < 0$) (Fig. 2A, panels A1 and A3). Experimental spike-trains demonstrated an average $\bar{\rho }_1 = -0.52 \pm 0.14$ ($\text {mean} \pm \text {s.d.}$) ($N = 52$). Nearly half of these fibers had $\rho _1 > -0.5$ ($N = 24$) while the remaining fibers had $\rho _1 < -0.5$ ($N = 28$). The second SCC ($\rho _2$), between every other ISI (Fig. 2A, panels A2 and A3) assumed positive ($N = 36$) or negative ($N = 16$) values with an average of $\bar{\rho }_2 = 0.10 \pm 0.18$. The correlation coefficient at the second lag ($\rho _2$) is linearly dependent on $\rho _1$ with a positive $\rho _2$ more likely if $\rho _1 < - 0.5$ (Fig. 2A, panel A3). The correlation between $\rho _1$ and $\rho _2$ is $-0.94$. The linear relationship is described by the equation $\rho _2 = -1.18 \rho _1 - 0.51$, with a standard error (SE) of 0.06 (slope) and 0.03 (intercept), and significance $p = 1.11 \times 10^{-25}$ (slope) and $p = 1.42 \times 10^{-21}$ (intercept). This is close to the line $\rho _2 = -\rho _1 - 0.5$, the significance of which is discussed further below. For all afferents across the population, Fig. 2B depicts $\rho _1$ as a function of the average firing rate of the neuron, and Fig. 2C depicts $\rho _1$ as a function of the coefficient of variation (CV) of the ISIs. Finally, the sum of the SCCs for each fiber taken over the first fifteen lags (excluding zeroth lag) has a population mean $\sum _k\,\rho _k = -0.475 \pm 0.042$ ($N = 52$) which is just short of $-0.5$ (Fig. 2D). Fifty out of 52 afferents had sums larger than $-0.5$, whereas the remaining two afferents had a sum smaller than $-0.5$ ($-0.504$ and $-0.502$, respectively). However, for these two fibers, the difference from $-0.5$ is small. We are not able to state with confidence that the trailing digits of these two estimates are significant.

**Fig. 3:**
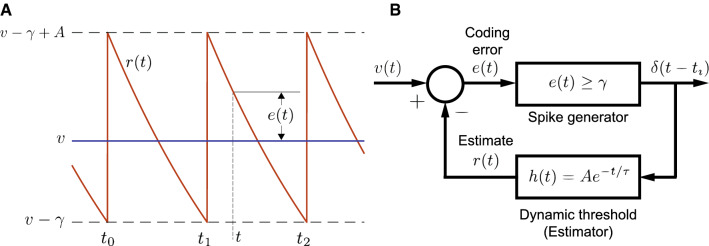
Schematic of neuron with deterministic dynamic threshold. **A**  *v* is a constant bias voltage that generates spontaneous activity, $r\left( t\right)$ is a dynamic threshold, $\gamma$ is a spike threshold such that a spike is emitted when $v-r\left( t\right) = \gamma$. Following a spike, the dynamic threshold suffers a jump of fixed magnitude *A*. The membrane voltage is non-resetting. Spike times are $t_0,\,t_1,\,t_2,\,\ldots$, etc. Historically, the spike threshold was set to zero, i.e., $\gamma = 0$ and a spike is fired when $v = r\left( t\right)$. Although it makes no difference to the results presented here, the more general form with $\gamma$ is considered for reasons entered in the discussion. **B** The spike encoder with dynamic threshold can be viewed as a feedback control loop where the spike-train $\delta \left( t - t_i\right)$ is filtered by $h\left( t\right)$ to generate the time-varying dynamic threshold $r\left( t\right)$ which can be viewed as an estimate of the membrane voltage *v*(*t*). The comparator generates the estimation error $e(t) = v-r\left( t\right)$ (see also Panel **A**). The estimation or coding error drives the spike generator, and a spike is fired whenever $e(t) \ge \gamma$. The simplest form for the dynamic threshold (the estimation filter) $h\left( t\right)$ mimics an RC membrane whose filter function is low-pass and given by $h\left( t\right) = A\exp \left( -t/\tau \right)$. This is the form shown in panel A

### Deterministic dynamic threshold model

In the simplest form of the dynamic threshold model (Jones et al. 2015), the spike-initiation threshold is a dynamic variable governed by two time-varying functions: the sub-threshold membrane potential *v*(*t*) and a time-varying or dynamic threshold $r\left( t\right)$ (Fig. 3A). In the sub-threshold regime, $v(t) < r\left( t\right)$. Most models (e.g., Kobayashi et al. 2009) assume that a spike is emitted when *v*(*t*) exceeds $r\left( t\right)$ from below. To induce memory, the dynamic threshold is never reset (Fig. 3A) but suffers a jump in value whenever a spike is generated and then gradually relaxes to its quiescent value. This “jump and decay” is a fixed function which is called the dynamic threshold, and it usually takes the form $h\left( t\right) = A\exp \left( -t/\tau \right)$ where *A* is the instantaneous jump in threshold following a spike at $t = 0$, and $\tau$ is the time-constant of relaxation. Between two spikes $t_k < t \le t_{k+1}$ the dynamic threshold is $r(t_k^-) + h(t - t_k)$ where $r(t_k^-)$ is the value assumed immediately before the spike at $t = t_k$. It captures the sum over the history of spiking activity.

Most neuron models in the literature, including models incorporating a dynamic threshold, typically integrate the input using a low-pass filter (i.e., pass it through a leaky integrator) or a perfect integrator and then reset the membrane voltage to a constant $v_r$ following a spike (e.g., Chacron et al. 2001; Schwalger and Lindner 2013). Some leaky integrators have been assumed to be non-resetting (e.g., Kobayashi et al. 2009). In the form of the model considered in this work and earlier (Jones et al. 2015; Johnson et al. 2015, 2016), the voltage *v*(*t*) is not integrated (i.e., there is no filtering), and it is not reset following a spike. These assumptions remove the effects of filtering in the soma and dendrites and remove all the nonlinearities except for the spike-generator, which we assume generates a sequence of Dirac-delta functions.

For modeling spontaneous activity, we consider a steady DC-level bias voltage $v > 0$. In its most general form, a spike is fired whenever $v - r\left( t\right) \ = \gamma$, where $\gamma$ is a constant spike-threshold (Fig. 3A). Historically, and in much of the literature $\gamma = 0$ (e.g., Kobayashi et al. 2009; Chacron et al. 2001; Schwalger and Lindner 2013). We use the more general form with a constant, nonzero $\gamma$. The specific value assumed by $\gamma$ plays a role in optimal estimation of the stimulus (Jones et al. 2015; Johnson et al. 2016) but does not influence serial correlation coefficients. This is addressed in the discussion. The major advantage of these simplifications is that they allow us to focus on the role of the dynamic threshold element $h\left( t\right)$ in generating SCCs.

To make explicit the presence of memory, we note that the condition for firing the *k*th spike at $t_k$ is met when

5 $$\begin{aligned} v - r\left( t_k\right) = v - (v - \gamma + A)\sum _{i = 1}^{k-1}\,\exp \left( -\frac{t_k - t_i}{\tau }\right) = \gamma . \nonumber \\ \end{aligned}$$

It is evident that memory builds up because of a summation over history of spiking activity. The spike encoder with dynamic threshold implicitly incorporates a feedback loop (Fig. 3B) and so a different view of the above model is to think of the dynamic threshold $r\left( t\right)$ as an ongoing estimate of the membrane voltage $v\left( t\right)$. Here, the dynamic threshold $h\left( t\right) = A\exp \left( -t/\tau \right)$ acts as a linear low-pass filter. It filters the spike train to form an ongoing estimate $r\left( t\right)$ of the voltage $v\left( t\right)$. The instantaneous error in the estimate (the coding error) is then $e\left( t\right) = v\left( t\right) - r\left( t\right)$ (Fig. 3B). When the error exceeds $\gamma$, a spike is output and the threshold increases by *A* to reduce the error. The time-varying dynamic threshold tracks $v\left( t\right)$ much like a home thermostat tracks a temperature set-point (Fig. 3A). Viewed in this way, it is the estimation error, and not the signal $v\left( t\right)$, which directly drives the spike generator and determines when the next spike should be generated (Fig. 3B).

From Fig 3A, we can approximate the exponential with a piece-wise linear equation when the ISI is small. If $t_{i-1}$ and $t_{i}$, $i \ge 1$, are successive spike-times (Fig. 3B), then the time evolution of the dynamic threshold $r\left( t\right)$ is given by

6 $$\begin{aligned} r\left( t\right)= & {} (v - \gamma + A)\,\exp (-\frac{t - t_{i-1}}{\tau }),\quad t_{i-1} < t \le t_{i}\,, \nonumber \\= & {} (v - \gamma + A)(1 - \frac{t - t_{i-1}}{\tau }) + {{\,\mathrm{\mathrm {O}}\,}}(\left( t - t_{i-1}\right) ^2). \end{aligned}$$

Note that *v*, $\gamma$, *A*, $\tau$ are constant, and so we can define $m = \left( v-\gamma +A\right) /\tau$ so that the slope of the decaying dynamic threshold is $-m$. The ISI can be obtained directly as (see Appendix for details)

7 $$\begin{aligned} t_{i} - t_{i-1} = \frac{A\tau }{v - \gamma + A} = \frac{A}{m} = \text {Constant}\,, \end{aligned}$$

which is the deterministic firing rule for a spike generator with a constant, DC-level input voltage.

### Stochastic extension of the dynamic threshold model

In the schematic shown in Fig. 3B, noise injected in the body of the feedback loop will reverberate around the loop and generate memory. In the literature, a stochastic extension of this model is usually $v -r\left( t\right) + w\left( t\right) = 0$, where *w* is independent Gaussian noise. That is, noise is continuous. Here, we consider a discrete, noisy threshold $\gamma$. Subsequently, we will provide some results for a noisy time-constant $\tau$ in the dynamic threshold element.

**Fig. 4:**
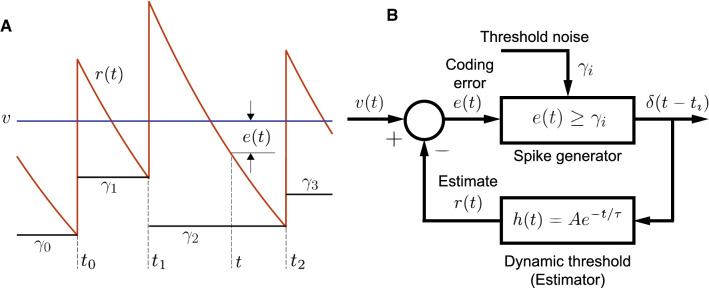
Schematic of a neuron with dynamic threshold and stochastic firing threshold. Panel descriptions follow Fig. 3 and only differences are noted. **A**  The spike threshold is $\gamma _i = v - r\left( t\right)$ where $\gamma _i$ is a random value generated at $t_{i-1}^+$, and held constant until the next spike at $t_i$. The discrete noise sequence $\gamma _i$ is generated by a discrete wide-sense stationary process with mean $\gamma$ and unknown discrete autocorrelation function ${{\,\mathrm{\mathbf {R}}\,}}_k$. The goal is to determine ${{\,\mathrm{\mathbf {R}}\,}}_k$ which will generate a prescribed sequence of ISI serial correlation coefficients $\rho _k$. To reduce clutter, the spike threshold $v - \gamma _i$ is depicted as $\gamma _i$. **B** Block diagram showing the stochastic modification of the spike threshold. Symbols and additional description as in text and Fig. 3

Figure 4 depicts the stochastic dynamic threshold model where the spike threshold ($\gamma$) is a stochastic process. All other aspects of the model are unchanged from the deterministic model (Fig. 3). Let $\gamma$ be a discrete wide-sense stationary process with mean ${{\,\mathrm{\mathbf {E}}\,}}[\gamma ]$, discrete autocorrelation function ${{\,\mathrm{\mathbf {R}}\,}}_k$ and power ${{\,\mathrm{\mathbf {E}}\,}}\left[ \gamma ^2\right] = {{\,\mathrm{\mathbf {R}}\,}}_0$. The spike threshold with additive noise assumes the random value $\gamma _i$, $i \ge 1$ immediately after the $(i-1)$th spike and remains constant in the time interval $t_{i-1} < t \le t_{i}$ (Fig 4A) (Chacron et al. 2004a; Gestri et al. 1980). Thus, the *i*th spike is emitted when the error satisfies the condition

8 $$\begin{aligned} e\left( t_i\right) \ge \gamma _i, \quad \text {for} \, t > t_{i-1}. \end{aligned}$$

Subsequently the instantaneous value of the dynamic threshold jumps to a higher value specified by $v - \gamma _i + A$, and the noisy spike threshold assumes a new value $\gamma _{i+1}$. From Fig. 4A proceeding as before

9 $$\begin{aligned} t_{i} - t_{i-1} = \frac{1}{m}\left\{ \gamma _{i} - \gamma _{i-1} + A\right\} . \end{aligned}$$

**Fig. 5:**
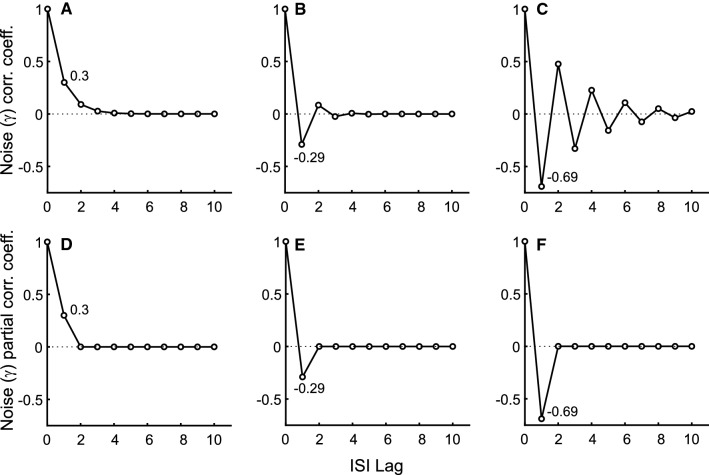
Threshold noise, $\gamma _k$: Normalized autocorrelation (top row, ${{\,\mathrm{\mathbf {R}}\,}}_k$) and partial autocorrelation functions (bottom row, calculated from ${{\,\mathrm{\mathbf {R}}\,}}_k$ and Eqs. 2, 3, and 4). **A** Generates Type I ISI SCCs matched to the afferent depicted in Fig. 1A with no bursting activity. **B** Generates Type II ISI SCCs from an afferent with moderate bursting activity. **C** Generates Type II ISI SCCs matched to the afferent depicted in Fig. 1B with strong bursting activity. Autocorrelation function that matches Type III SCCs will be zero for all nonzero lags (not shown). Panels **D**–**F** show partial autocorrelation functions of the corresponding noise process shown in panels (**A**–**C**), respectively. The partial autocorrelations are nonzero for the first lag, and zero for all lags $k > 1$. By design, the noise generator is a first-order autoregressive (AR) process (see Eqs. 18 and 22 for ${{\,\mathrm{\mathbf {R}}\,}}_k$)

The mean ISI is therefore ${{\,\mathrm{\mathbf {E}}\,}}\left[ t_{i}-t_{i-1}\right] = A/m$ as in the deterministic case given by Eq. (7). From the assumption of wide-sense stationarity, the autocorrelation function ${{\,\mathrm{\mathbf {E}}\,}}\left[ \gamma _i\, \gamma _j\right]$ can be written as ${{\,\mathrm{\mathbf {R}}\,}}\left( t_i - t_j\right)$. This is a discrete autocorrelation function whose discrete nature is made clear in the following way (see also Appendix). Denote the mean of the *k*th-order interval by $T_k = {{\,\mathrm{\mathbf {E}}\,}}[t_{i+k} - t_i]$, then the mean ISI is $T_1$ $(=A/m)$, and further $T_k = kT_1$. A realization of the random variable $\gamma$ is generated once every ISI and thus, ${{\,\mathrm{\mathbf {R}}\,}}$ takes discrete values at successive multiples of the mean ISI, i.e., ${{\,\mathrm{\mathbf {R}}\,}}\left( T_1\right)$, ${{\,\mathrm{\mathbf {R}}\,}}\left( T_2\right)$, etc., and will be denoted by ${{\,\mathrm{\mathbf {R}}\,}}_1$, ${{\,\mathrm{\mathbf {R}}\,}}_2$, etc., respectively. As noted before, ${{\,\mathrm{\mathbf {R}}\,}}_0$ is noise power. That is, we can write

10 $$\begin{aligned} {{\,\mathrm{\mathbf {R}}\,}}\left( t_{i+k} - t_i\right) = {{\,\mathrm{\mathbf {R}}\,}}\left( T_k\right) = {{\,\mathrm{\mathbf {R}}\,}}_k\,, \end{aligned}$$

where *k* is the number of intervening ISIs. The serial correlation coefficients at lag *k* are defined as (Cox and Lewis 1966)

11 $$\begin{aligned} \rho _k = \frac{{{\,\mathrm{\mathrm {Cov}}\,}}\left( \left( t_{i}-t_{i-1}\right) ,\,\left( t_{i+k}-t_{i+k-1}\right) \right) }{{{\,\mathrm{\mathrm {Var}}\,}}\left( t_{i}-t_{i-1}\right) ^{1/2}{{\,\mathrm{\mathrm {Var}}\,}}\left( t_{i+k}-t_{i+k-1}\right) ^{1/2}}. \end{aligned}$$

For a wide-sense stationary process, the covariances are constant, so the subscript *i* can be dropped. From the relations and definitions in Eqs. (9), (10), and (11), and after some routine algebra (see Appendix), we obtain

12 $$\begin{aligned} \rho _0= & {} 1\,, \end{aligned}$$

13 $$\begin{aligned} \rho _k= & {} -\frac{{{\,\mathrm{\mathbf {R}}\,}}_{k-1} - 2{{\,\mathrm{\mathbf {R}}\,}}_{k} + {{\,\mathrm{\mathbf {R}}\,}}_{k+1}}{2\left( {{\,\mathrm{\mathbf {R}}\,}}_0 - {{\,\mathrm{\mathbf {R}}\,}}_1\right) }\,, \quad \ k \ge 1. \end{aligned}$$

Further below we determine the ${{\,\mathrm{\mathbf {R}}\,}}_k$ from experimental data. The serial-correlation coefficients given by Eqs. (12) and (13) are independent of the slope *m* of the decay rate of the dynamic threshold, and its gain *A*. Thus, for a constant input the observed correlation structure of the spike-train is determined solely by the noise added to the deterministic firing threshold $\gamma$.

#### Limiting sum of ISI correlation coefficients and power spectra

We make the assumption that the process $\gamma$ is aperiodic and the autocorrelation function ${{\,\mathrm{\mathbf {R}}\,}}_N \rightarrow 0$ when $N \rightarrow \infty$. That is, the noise process decorrelates over long time-scales. From this assumption and Eq. (13), it follows that (see Appendix)

14 $$\begin{aligned} \sum _{k=1}^{\infty } \rho _k = -\frac{1}{2}. \end{aligned}$$

This is the limiting sum of ISI serial correlation coefficients. Let the mean and variance of ISIs be denoted by $T_1$ and $V_1$, respectively, and the coefficient of variation of ISIs as $C = \sqrt{V_1}/T_1$. If $P\left( \omega \right)$ is the power spectrum of the spike train, then the DC-component of the power is given by (Cox and Lewis 1966)

15 $$\begin{aligned} \underset{\omega \rightarrow 0}{\lim }\,P\left( \omega \right) = \frac{C^2}{2\pi T_1} \left( 1 + 2\sum _{k=1}^{\infty }\,\rho _i\right) . \end{aligned}$$

Introducing Eq. (14) into Eq. (15), we obtain

16 $$\begin{aligned} \underset{\omega \rightarrow 0}{\lim }\,P\left( \omega \right) = 0\,, \end{aligned}$$

yielding a perfect DC block.

The limiting sum of SCCs from experimental spike trains, with the sum calculated up to 15 lags, is depicted in Fig. 2D. The population of afferents demonstrated a range of limiting sums with mean sum of $-0.475 \pm 0.04$. As noted earlier, the limiting sum was less than $-0.5$ in only two afferents, where the sums were estimated as $-0.505$ and $-0.502$.

#### Predicting Type I and Type II serial correlation coefficients

In the relationships for the SCCs given by Eqs. (12) and (13), the noise process that drives the spike generator has an unknown correlation function ${{\,\mathrm{\mathbf {R}}\,}}$ which must be determined so as to predict experimentally observed SCCs. Here, we are interested in identifying the simplest process that can satisfactorily capture observed SCCs.

Consider a Gauss–Markov, i.e., Ornstein–Uhlenbeck (OU) process which relaxes as $\exp \left( -t/\tau _\gamma \right)$ with relaxation time $\tau _\gamma$, where $\gamma$ signifies the spike threshold. We are interested in a discrete-time realization of the process. We first convert the decaying exponential to a geometric series where the time-base is in integer multiples of the mean ISI (see Sect. 3.3 and Eq. 10). Thus, the mean ISI becomes the “sampling period” and the exponential can be sampled at multiples of $T_1$, i.e., $t = kT_1$, with $k = 1,\,2,\,3,\ldots$. Following this procedure, the continuous-time relaxation process $\exp \left( -kT_1/\tau _\gamma \right)$ transforms to a discrete-time (sampled) geometric series with $a^{-k} = \exp \left( -kT_1/\tau _\gamma \right)$, where the parameter $a = \exp \left( -T_1/\tau _\gamma \right) < 1$. In the discrete formalism, the continuous-time first-order Gauss–Markov process (OU process) with relaxation time $\tau _\gamma$, transforms to a first-order autoregressive (AR) process with parameter *a*. We show this below, where we define two first-order AR processes which will generate Type I and Type II SCCs, respectively.

*Type I serial correlation coefficients* Type I afferent spiking activity demonstrates serial correlations where $-0.5< \rho _1 < 0$, and subsequent $\rho _k$ are negative and diminish to 0 with increasing *k* (Fig. 1A). These spike trains have a unimodal ISI and do not display bursting activity. They can be generated from the ansatz, a first-order AR process

17 $$\begin{aligned} \gamma _k = a \gamma _{k-1} + w_k, \end{aligned}$$

where $\gamma _k$ is the noise added to the threshold at the *k*th spike (Fig. 4), *a* is the relaxation time of the discrete noise process (see above), and $w_k$ is white noise input to the AR process. The output $\gamma$ is wide-sense stationary, and its discrete autocorrelation function ${{\,\mathrm{\mathbf {R}}\,}}_k$ is

18 $$\begin{aligned} {{\,\mathrm{\mathbf {R}}\,}}_k = \frac{a^k}{1-a^2}. \end{aligned}$$

Equations (13) and (18) yield the geometric series

19 $$\begin{aligned} \rho _k = -\frac{a^{k-1}\left( 1 - a\right) }{2}\,, \quad k \ge 1. \end{aligned}$$

From Eq. (19), and noting that $0< a < 1$, we conclude that $\rho _1 > -0.5$, $\rho _k < 0$ for all *k*, $\mid \rho _{k-1}\mid > \mid \rho _k\mid$, and $\rho _k \rightarrow 0$ as $k \rightarrow \infty$. This is the observed Type I pattern of SCCs. Further, summing the geometric series Eq. (19) yields $\sum _{k \ge 1}\,\rho _k = -0.5$ as stated in Eq. (14). The AR parameter *a* can be estimated from experimentally determined SCCs. From Eq. (19) this is

20 $$\begin{aligned} a = 1 +2\rho _1\,. \end{aligned}$$

In practice, a better estimate is often obtained from the ratio $a = \rho _2/\rho _1$, where $\rho _k$ are available from experimental data.

**Fig. 6:**
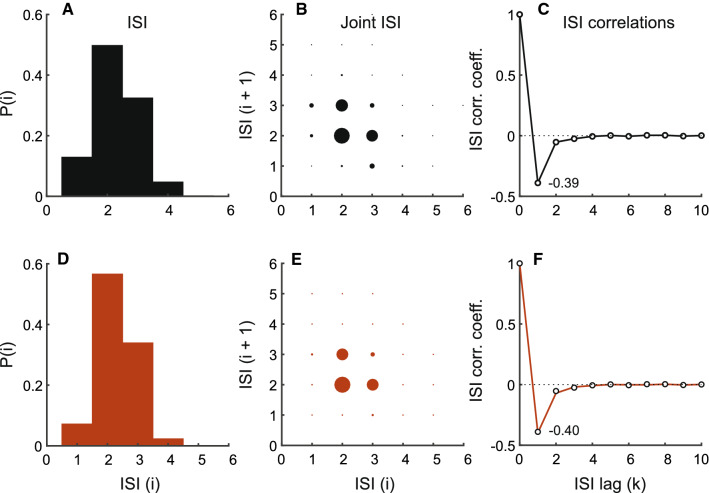
Type I neuron with no bursting activity. Top row depicts experimental spike-train from a P-type primary electrosensory afferent. **A** The spike train has a unimodal interspike interval distribution (ISI) and does not display bursting activity. Abscissa is ISI in multiples of electric organ discharge (EOD) period and ordinate is probability. **B** Joint interspike interval distribution showing probability of observing successive intervals $\text {ISI}\left( i\right)$ (abscissa) and $\text {ISI}\left( i+1\right)$ (ordinate). The size of the circle is proportional to the probability of jointly observing the adjacent ISIs, i.e., $P\left( i, \, i+1\right)$. **C** Normalized correlation (ordinate) as a function of lag measured in multiples of mean ISI (abscissa). Spike-train sampled at EOD period. ISI correlations for this afferent are shown in Fig. 1A at a different sampling rate (see Methods). Bottom row depicts results for matched model using colored noise with correlation function given by Eq. (18) and shown in Fig. 5A. Panel descriptions as in top row, except in F where open circles denote experimental data taken from (**C**). EOD period: 1.06 ms, mean ISI: 2.42 ms. To generate model results, $v = 1.845$ V, adaptive threshold parameters: $A = 0.15$ and $\tau = 30$ ms, AR parameter: $a = 0.4$ ($\tau _\gamma = 2.09$ ms), and ${{\,\mathrm{\mathbf {R}}\,}}_0 = 1.07 \times 10^{-3}~\text {V}^2$ ($\text {SNR} = 35~\text {dB}$)

We model a Type I P-type primary electrosensory afferent depicted in Fig. 1A using a noisy threshold with correlation function ${{\,\mathrm{\mathbf {R}}\,}}_k$ specified by Eq. (18) and shown in Fig. 5A. The dynamic threshold parameters were determined from the experimental data, and tuned so that they matched the afferent SCCs, ISI, and joint distributions (Fig. 6). By design, the noise process is first-order autoregressive, and this is reflected in the partial autocorrelation function calculated from the noise samples (Fig. 5D). The top row (Fig. 6A–C) depicts the ISI distribution, joint ISI distribution, and the serial correlation coefficients, respectively. Type I spike trains do not display bursting activity and their ISI distribution is unimodal. The bottom row (Fig. 6D–F) shows data from a matched model using noise correlation function given by Eq. (18). SCCs of adjacent ISIs $\rho _1$ are $-0.39$ (data) and $-0.40$ (model). The mean sum of SCCs are $-0.48$ (data) and $-0.5$ (model). Thus, the observed pattern of Type I SCCs is reproduced.

*Type II serial correlation coefficients* Type II afferent spiking activity demonstrates serial correlations where $\rho _1 < -0.5$ and successive $\rho _k$ alternate between positive ($\rho _{2k})$ and negative ($\rho _{2k+1}$) values, are progressively damped, and diminish to zero (Fig. 1B). These spike trains have bimodal ISIs and display bursting activity. Proceeding as before, they can be generated from the ansatz, a first-order AR process

21 $$\begin{aligned} \gamma _k = -a \gamma _{k-1} + w_k, \end{aligned}$$

with discrete autocorrelation function

22 $$\begin{aligned} {{\,\mathrm{\mathbf {R}}\,}}_k = \frac{(-a)^k}{1-a^2}\,. \end{aligned}$$

Equations (13) and (22) yield the geometric series

23 $$\begin{aligned} \rho _k = -\frac{(-a)^{k-1}\left( 1 + a\right) }{2}\,, \quad k \ge 1\,. \end{aligned}$$

From Eq. (23), and noting that $0< a < 1$, we conclude that $\rho _1 < -0.5$, $\rho _{2k} > 0$, $\rho _{2k+1} < 0$, $\mid \rho _{2k}\mid > \mid \rho _{2k+1}\mid$, and $\rho _k \rightarrow 0$ as $k \rightarrow \infty$. This is the observed Type II pattern of SCCs. Further, summing the geometric series (Eq. 23) yields $\sum _{k \ge 1}\,\rho _k = -0.5$ as stated in Eq. (14). The AR parameter *a* can be estimated from experimentally determined SCCs. From Eq. (23) this is

24 $$\begin{aligned} a = -(1 +2\rho _1)\,. \end{aligned}$$

As noted for Type I SCCs a better estimate is often obtained from the ratio $a = -\rho _2/\rho _1$. Finally, we note that the Type I formalism extends nicely to the Type II formalism with the only difference being that the coefficient of the first-order process (*a*) becomes negative. This simple substitution in Eqs. (18), (19), and (20) will result in Eqs. (22), (23), and (24), respectively

**Fig. 7:**
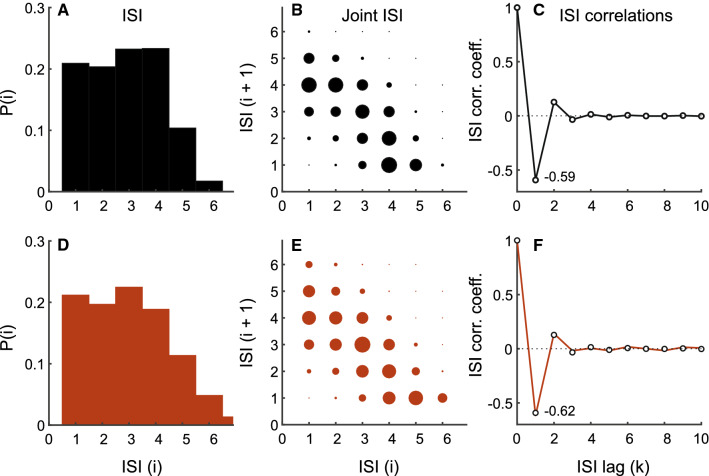
Type II neuron showing moderate bursting activity. Description is identical to Fig. 6. Top row depicts experimental spike train from a P-type primary electrosensory afferent. Bottom row depicts results for matched model using colored noise with correlation function given by Eq. (22) and shown in Fig. 5B. EOD period: 1.31 ms, mean ISI: 3.77 ms. To generate model results, $v = 1.845$ V, adaptive threshold parameters: $A = 0.28$ and $\tau = 26$ ms, AR parameter: $a = 0.29$ ($\tau _\gamma = 3.18$ ms), and ${{\,\mathrm{\mathbf {R}}\,}}_0 = 9.22 \times 10^{-3}~\text {V}^2$ ($\text {SNR} = 26~\text {dB}$)

**Fig. 8:**
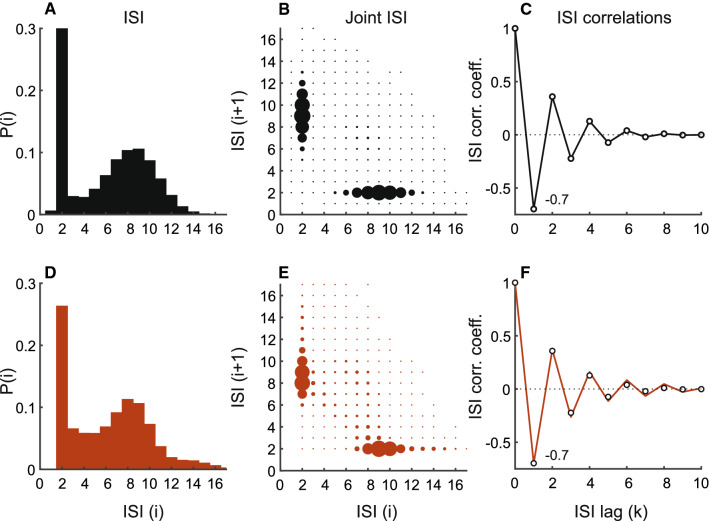
Type II neuron showing strong bursting activity. Description is identical to Fig. 6. Top row depicts experimental spike train from a P-type primary electrosensory afferent. SCCs for this afferent are shown in Fig. 1B at a different sampling rate (see Methods). Bottom row depicts results for matched model using colored noise with correlation function given by Eq. (22) and shown in Fig. 5C. EOD period: 1.04 ms, mean ISI: 6.04 ms. To generate model results, $v = 1.845~\text {V}$, adaptive threshold parameters: $A = 0.19$ and $\tau = 60$ ms, AR parameter: $a = 0.69$ ($\tau _\gamma = 16.89$ ms), and ${{\,\mathrm{\mathbf {R}}\,}}_0 = 6.4 \times 10^{-3}~\text {V}^2$ ($\text {SNR} = 27~\text {dB}$)

We model Type II P-type primary electrosensory afferents using a noisy threshold with correlation function ${{\,\mathrm{\mathbf {R}}\,}}_k$ specified by Eq. (22). The noise correlation function and dynamic threshold parameters were determined from the experimental data, and tuned so that they matched the afferent SCCs, ISI, and joint distributions. We demonstrate with two examples: (i) moderate bursting activity and (ii) strong bursting activity. The moderately bursting neuron has a broad bimodal ISI distribution (Fig. 7A–C), and its ISI and joint ISI distributions, and SCCs are captured by the matched model (Fig. 7D–F). The noise correlation function for generating the model spike-train is depicted in Fig. 5B, and the noise partial correlation function in Fig. 5E. SCCs of adjacent ISI $\rho _1$ are $-0.59$ (data) and $-0.62$ (model). The mean sum of SCCs are $-0.49$ (data) and $-0.5$ (model). The strongly bursting neuron has a well-defined bimodal distribution (Fig. 8A–C), and its ISI and joint ISI distributions, and SCCs are captured by the matched model (Fig. 8D–F). The noise correlation function for generating the model spike-train is depicted in (Fig. 5C), and the noise partial correlation function in Fig. 5F. SCCs of adjacent ISI $\rho _1$ are $-0.7$ (data) and $-0.7$ (model). The mean sum of SCCs are $-0.49$ (data) and $-0.5$ (model). Thus, in both cases (Figs. 7 and 8), the observed patterns of Type II SCCs are reproduced. All observed afferent spike trains were either Type I or Type II.

We mention in passing that the noise correlation functions depicted in Fig. 5A–C should not be confused with the SCCs depicted in panels C and F in Figs. 6, 7, and 8.

*Type III serial correlation coefficients* Type III afferent spiking activity demonstrates serial correlations where $\rho _1 = -0.5$ and all $\rho _k = 0$ for $k \ge 2$. This is a degenerate case, resulting in a singleton with a unique set of SCCs. Such spike trains can be generated by a process where spike threshold noise is uncorrelated, and hence white. In this case, ${{\,\mathrm{\mathbf {R}}\,}}_0 = \sigma _\gamma ^2$ is noise power, and ${{\,\mathrm{\mathbf {R}}\,}}_k = 0$ for $k \ge 1$. We see immediately from Eqs. (12) and (13) that the $\rho _k$ have the prescribed form. Further, we note that trivially $\sum _{k \ge 1}\,\rho _k = -0.5$ as stated in Eq. (14). We mention in passing, and for later discussion, that white noise added to the spike threshold is equivalent to setting $a = 0$ ($\tau _\gamma = 0$) in the first-order AR process defined in Eq. (17). A spike-train with exactly Type III SCCs has not been observed in the experimental data presented here.

#### Partial autocorrelation functions

**Fig. 9:**
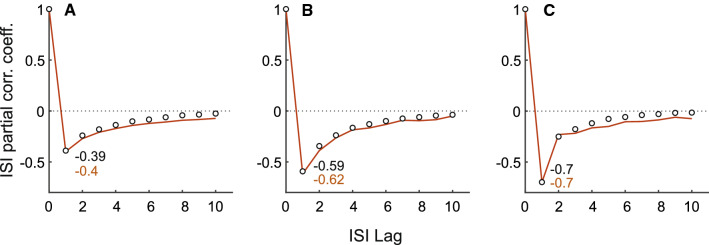
Normalized ISI partial autocorrelation functions (PACFs) estimated from afferent ISI sequences (open circles) and models (red line). **A**–**C** depict the PACFs for the three example afferents shown in Figs. 6, 7, and 8, respectively. These PACFs should be compared with the normalized ISI autocorrelation function (ACF) generated for the same data (Figs. 6F, 7F, and 8F, respectively). **A**. Type I, non-bursting (ACFs shown in Fig. 6F). **B**. Type II, moderately bursting (ACFs shown in Fig. 7F). **C**. Type II, strongly bursting (ACFs shown in Fig. 8F). The first partial correlation coefficient $\phi _{1,\,1}$ is reported in each panel (data: black text, model: red text) and is the same as $\rho _1$ (Eq. 4). The long tails observed in the PACFs and ACFs suggest that the afferent and model spike-trains are not an AR process, but may be an autoregressive moving average (ARMA) process of unknown order

The autocorrelation function (ACF) at lag *k* includes the influence of the variable $X_{i+k}$ on $X_i$, and the influence of all the intervening lags $r < k$. The direct effect of $X_{i+k}$ on $X_i$ is not explicitly known. On the other hand, the partial autocorrelation (PAC) between two variables in a sequence $X_i$ and $X_{i+k}$ removes the linear influence of the intervening variables $X_{i+1},\,\ldots ,\,X_{i+k-1}$ (Box and Jenkins 1970). The PAC as a function of *k* is the PAC function or PACF. Section 2.2.2 in Methods discusses the differences between the ISI PACF and the ISI ACF with regard to AR and MA processes. We estimated the PACF from experimental data and the modeled spike trains using Eqs. (2), (3), and (4). These are the same model spike trains used in the calculation of the ACFs. The PACFs are shown in Fig. 9A–C for the afferents shown in Figs. 6, 7, and 8, respectively. By definition the first partial autocorrelation $\phi _{1,\,1} = \rho _1$. In contrast to the SCCs where correlation coefficients $\rho _k$ could be positive or negative for $k \ge 2$, all partial autocorrelations are negative irrespective of the type of afferent.

#### Dynamic threshold with a random time-constant

**Fig. 10:**
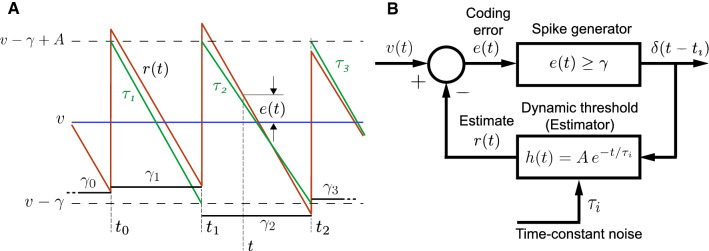
Dynamic threshold with a stochastic filter time-constant. **A**. Geometry of the spiking mechanism. The input is a deterministic piece-wise constant signal (*v*, here shown to be DC-valued). Two equivalent spike-generation processes are possible: Case (i) The spike threshold $\gamma$ is noisy, while the dynamic threshold filter *h*(*t*) has fixed time-constant $\tau$. This is the same as Fig. 4A. The spike threshold is a constant term $v - \gamma$ plus a random perturbation $x_i$ generated at $t_{i-1}^+$, and held constant until the next spike at $t_i$ (black). The time-varying dynamic threshold *h*(*t*) is shown in red. Case (ii) The function *h*(*t*) shown in green has a random time-constant with mean $\tau$ while the spike threshold is $v - \gamma$, and is constant. Threshold noise $x_i$ from Case (i) can be transformed into a noisy dynamic threshold time-constant $\tau _i$ which is fixed between spikes, and takes correlated values over successive ISIs. Spike-times for both cases are identical. **B** Block diagram illustrating the feedback from the estimator that generates optimal spike times. The time-constant $\tau$ is a random variable that is constant between spikes, and is correlated over interspike intervals (ISIs). It is of interest to ask what is the relationship between the correlation functions in the two cases. Symbols and additional description as in text

The dynamic threshold has three parameters *A*, $\gamma$, and $\tau$. We have so far described a stochastic model based on a noisy $\gamma$ which is formally equivalent to a noisy *A*. We now consider a stochastic dynamic threshold model based on a noisy time-constant $\tau$. We can transform the adaptive threshold model with a random spike threshold from the previous section and Fig. 4 so that the time-constant of the adaptive threshold filter $h\left( t\right)$ is a random variable with mean $\tau$ (Fig. 10). From Eq. (9) the random variate $\gamma _i - \gamma _{i-1}$ which appears in the time-base can be transformed into the random variate $m_i$ which is the slope of the linearized adaptive threshold (green line, Fig. 10). From

25 $$\begin{aligned} m_i = \frac{\tau _i}{v - \gamma +A}\,, \end{aligned}$$

we obtain

26 $$\begin{aligned} A\tau _i = \tau \left\{ \gamma _{i} - \gamma _{i-1} + A\right\} , \end{aligned}$$

and so, ${{\,\mathrm{\mathbf {E}}\,}}\left[ \tau _i\right] = \tau$. As noted, this is the mean value of the dynamic threshold filter time-constant. It is immediately apparent from Eqs. (9) and (26) that the covariance of the sequence $\tau _i$ (sampled at the ISIs) is the same as the covariance of the ISI sequence up to a scale-factor, and therefore the serial correlations of $\tau$ ($\rho _{\tau ,\,k}$, $k \ge 0$) are the same as the serial correlations of the ISIs. Therefore (see Appendix for details)

27 $$\begin{aligned} \rho _{\tau ,\,0}= & {} 1, \end{aligned}$$

28 $$\begin{aligned} \rho _{\tau ,\,k}= & {} -\frac{{{\,\mathrm{\mathbf {R}}\,}}_{k-1} - 2{{\,\mathrm{\mathbf {R}}\,}}_{k} + {{\,\mathrm{\mathbf {R}}\,}}_{k+1}}{2\left( {{\,\mathrm{\mathbf {R}}\,}}_0-{{\,\mathrm{\mathbf {R}}\,}}_1\right) }, \quad \ k \ge 1. \end{aligned}$$

The right side of the above equations, Eqs. (27) and (28), are the same as Eqs. (12) and (13), the expressions for the SCCs of a spike train generated with a noisy adaptive threshold. Thus, the random filter time-constants have the same serial correlation coefficients as the interspike intervals, $\rho _{\tau ,\,k} = \rho _k$. This is a “pass through” effect where the correlations observed in the time-constant are directly reflected in the ISI correlations.

In summary, a noisy threshold $\gamma$ or a noisy filter time-constant $\tau$ can be used to generate spike trains which have prescribed ISI SCCs. We have generated spike trains using a noisy threshold and will not duplicate the results for a noisy time-constant.

## Discussion

### Experimental observations

All experimentally observed P-type spike-trains from *Apteronotus leptorhynchus* demonstrated negative correlations between adjacent ISIs (Figs. 1, 2A). Thus, negative SCCs between adjacent ISIs may be an obligatory feature of neural coding, at least in this species. The implications for coding are discussed further below. A broad experimental observation is the roughly equal division of spike-trains into units with $\rho _1 > -0.5$ (non-bursting or Type I units, Fig. 1A, $N = 24$) and units with $\rho _1 < -0.5$ (bursting or Type II units, Fig. 1C, $N = 28$). Using a different method of classification, Xu et al. (1996) reported 31% of 117 units as bursting.

As a function of the firing rate, the first SCC, $\rho _1$ (Fig. 2C) shows a minimum at a firing rate of about 190 spikes/s with a V-shaped envelope which appears to set a lower bound on $\rho _1$ at low and high firing rates. At high firing rates, the mean ISI is small, and there is limited variability below the mean ISI because an ISI cannot be less than zero. This possibly increases $\rho _1$ (toward zero) as firing rate increases, and creates the effect shown in (Fig. 2C). Supporting this finding is the inverse relationship between $\rho _1$ and the coefficient of variation (CV) of ISI (Fig. 2C). Although the correlation is weak ($r = -0.67$) a large $\rho _1$ is more likely when the ISI CV is small. An explanation for the shape of the envelope on the low-firing rate flank in Fig. 2C is not readily apparent.

For Type I units $\rho _2 < 0$, whereas for Type II units $\rho _2 > 0$ (Fig. 2C). The former give rise to over-damped SCCs which remain negative and diminish to zero, while the latter give rise to under-damped or oscillatory SCCs which also diminish to zero. The dependence of $\rho _2$ on $\rho _1$ is linear for the most part and follows the equation $\rho _2 = -1.18\rho _1 - 0.51$ (Fig. 2C). The limiting sum of SCCs $\sum _{k \ge 1}\rho _k$ is close to $-0.5$ irrespective of the type of SCC pattern (Fig. 2D) (Ratnam and Goense 2004). With the exception of two afferent fibers, the sum of SCCs for the fibers was never smaller than $-0.5$, i.e., it was almost always true that $\sum _{k \ge 1}\rho _k \ge -0.5$. In two cases the sums were less than the limit ($-0.505$ and $-0.502$), possibly due to estimation error. Although the dominant SCCs are $\rho _1$ and $\rho _2$, their sum $\rho _1 + \rho _2$ is not close to $-0.5$ (i.e., $\rho _2 \ne -\rho _1 - 0.5$) and deviates, as stated above, with a linear relationship which follows $\rho _2 = -1.18\rho _1 - 0.51$. Thus, more terms (lags) are needed to bring the sum of SCCs close to the limiting value, and this results in significant correlations extending over multiple lags (time-scales). As discussed in an earlier work (Ratnam and Nelson 2000), long-short (short-long) ISIs create memory in the spike-train and keep track of the deviations of successive ISIs from the mean ISI ($T_1$). These deviations are a series of “credits” and “debits” which may not balance over adjacent ISIs, but will eventually balance so that a given observation of *k* successive ISIs returns to the mean with $t_k - t_0 \approx kT_1$. Such a process will exhibit long-range dependencies that may not be captured by SCCs.

That the dependencies may extend over multiple ISIs is confirmed from an analysis of joint dependencies of intervals extending to high orders (van der Heyden et al. 1998; Ratnam and Nelson 2000). All 52 units in *Apteronotus leptorhynchus* were at least second-order Markov processes, with about half ($N = 24$) being fifth-order or higher (Ratnam and Nelson 2000). Further, SCCs were not correctly predicted when only the adjacent ISI dependencies were preserved, i.e., were considered to be first-order Markov (Poggio and Viernstein 1964; Rodieck 1967; Ratnam and Nelson 2000). Indeed, an examination of the sequence of SCCs provides no indication of the extent of memory. For instance, short-duration correlations do not necessarily imply that ISI dependencies are limited to fewer adjacent intervals. Long-duration dependencies may be present even when the correlation time is short (van der Heyden et al. 1998). Conversely, a first-order Markov process produces a ringing in the serial correlogram ($\rho _k = \rho _1^k$) that can continue for ISIs much longer than two adjacent ISIs (Cox and Lewis 1966; Nakahama et al. 1972). In fact, for some P-type electrosensory afferent spike trains, the observed ISIs exhibited SCCs whose magnitudes were smaller than the SCCs for the matched first-order Markov model even though the experimental data were at least second-order or higher (see Fig. 8, Ratnam and Nelson 2000).

The stochastic process which generates ISIs may be more complex than a simple autoregressive (AR) or moving average (MA) process because afferent ISI correlation functions (Figs. 6F, 7F, and 8F) and partial autocorrelation functions (Fig. 9) are infinite in duration (see Methods for a distinction between the two functions, Box and Jenkins (1970)). This suggests that a more general ARMA process may be responsible for the generation of ISIs. The source of the mix of AR and MA processes is discussed further below when we consider the generating model.

### The dynamic threshold model

The experimental observations and dependency analyses motivated us to ask whether we could develop a stochastic model to reproduce a prescribed sequence of SCCs $\rho _1\,,\rho _2\,,\ldots \,,\rho _k$. We adopt a widely used and physiologically plausible model with a time-varying, i.e., dynamic, threshold. This is a simple model without much complexity, and has few parameters. The model allows us to probe patterns of SCCs as a function of these parameters. Further, it tests the extent to which we can describe experimental data with a simple model. Dynamic threshold models typically have three components: (1) dynamics of membrane voltage *v*(*t*) in response to an input signal, (2) a spike or impulse generator, and (3) a time-varying dynamic threshold *r*(*t*) which is elevated following a spike and subsequently relaxes monotonically. A spike is fired when the membrane voltage meets the (relaxing) threshold, thus forming a feedback loop (Fig. 3B). Models of adaptation with an adaptation current are broadly similar but instead of altering the threshold directly, they use an outward current to induce refractoriness (see, e.g., Benda et al. 2010; Benda and Herz 2003; Schwalger and Lindner 2013). In many models, the feedback loop is implicitly defined (as in conductance-based models), but it may also be explicitly defined as is done here so that $v(t) - r(t)$ reaches a fixed spike-initiation threshold. This threshold is usually taken to be zero, i.e., $v(t) = r(t)$ (Kobayashi et al. 2009), but here we assume it to be a nonzero value ($\gamma = A/2$) following Jones et al. (2015). This choice does not alter model behavior or the analyses presented here (see Methods) but has relevance to optimal coding and is discussed further below.

Models that do not utilize a dynamic threshold or an adaptation current are not discussed here because they are outside the scope of this work. The reader may consult Longtin et al. (2003), Lindner and Schwalger (2007), Farkhooi et al. (2009), Urdapilleta (2011) for more details and references to such models. Most dynamic threshold models that address negative correlations assume some form of perfect or leaky integrate-and-fire dynamics for the first two components listed above (Chacron et al. 2000, 2001, 2004b; Brandman and Nelson 2002; Benda et al. 2010), and an exponentially decaying dynamic threshold without spike reset. Noise is added to the time-varying threshold or to the input current and this results in negative serial correlations. Model complexity has been a major drawback in determining the precise role of noise in shaping ISI correlations (see, for instance, observations made in Chacron et al. 2004b; Lindner et al. 2005). Resets, hard refractory periods, sub-threshold dynamics due to synaptic filtering, and sometimes multiple sources of noise obscure the effects of signal propagation through the system and obscure signal dependencies. Thus, with few exceptions (see below), dynamic threshold models and models with adaptation currents, have been qualitative. They demonstrate some features of experimentally observed ISI distributions, and at best correlations between adjacent ISIs (i.e., $\rho _1$) (Geisler and Goldberg 1966; Chacron et al. 2000, 2003). On the other hand, reduced model complexity can result in a lack of biophysical plausibility. Thus a judicious choice of models should expose desired mechanisms while retaining enough important features of the phenomena.

### Model results

In recent years, deterministic dynamic threshold models with an exponential kernel have been used to predict spike-times from cortical and peripheral neurons (Kobayashi et al. 2009; Fontaine et al. 2014; Jones et al. 2015) (see Gerstner and Naud 2009, for an early review) and predict peri-stimulus time histograms (Jones et al. 2015; Johnson et al. 2016) with good accuracy. Capturing spike-times accurately is perhaps the first requirement in our analysis, and this gives confidence that the model may tell us something about ISI correlations. We eliminated sub-threshold dynamics and resets so that there is only one nonlinear element, the spike generator (Jones et al. 2015; Johnson et al. 2016). These are not serious restrictions, and they make the analysis tractable. We follow the usual practice of representing the dynamic threshold element with an exponential decay with time-constant $\tau$. The absence of reset implies that the time-varying dynamic threshold which carries memory is a simple convolution of the spike train with an exponential kernel $Ae^{-t/\tau }$ (Fig. 3B). We inject noise precisely in one of two places, either to perturb the spike threshold $\gamma$ (Fig. 4) or perturb the time-constant $\tau$ (Fig. 10). The two forms of perturbation are formally equivalent (see Appendix). We linearize the exponential so that we can obtain analytical solutions of SCCs. This is applicable at asymptotically high spike-rates (Jones et al. 2015; Johnson et al. 2015) and applies well to P-type afferent spike trains because of their high baseline firing rates, about 250-300 spikes/s (Bastian 1981; Xu et al. 1996; Ratnam and Nelson 2000). To fit ISI and joint-ISI distributions of individual P-type afferents, the parameters of the dynamic threshold element $h\left( t\right)$ (*A* and $\tau$) are obtained from the afferent spike-train (Jones et al. 2015). A single noise parameter, *a*, independent of the dynamic threshold, is obtained from the observed SCCs. These model elements and procedures allow us to determine the shaping of ISI correlations. The major results are

1. ISI correlations are determined by the autocorrelation function, ${{\,\mathrm{\mathbf {R}}\,}}$, of the noise process (Eqs. 12–13).
2. Non-bursting units and bursting units are described by the same functional relationship between ISI SCCs and ${{\,\mathrm{\mathbf {R}}\,}}$ (Eqs. 12–13).
3. Non-bursting spike trains (with unimodal ISI distribution) are generated by slow noise with a decaying (positive) correlation function ${{\,\mathrm{\mathbf {R}}\,}}$, which in its simplest form is given by Eq. (18) (e.g., Fig. 6).
4. Bursting spike trains (with bi-modal ISI distribution) are generated by fast noise with an oscillating correlation function ${{\,\mathrm{\mathbf {R}}\,}}$, which in its simplest form is given by Eq. (22) (e.g., Figs. 7 and 8).
5. The two types of correlation functions are described by the sign of a single parameter, the parameter *a* of a first-order autoregressive process (Eqs. 21 and 17). The AR parameter is directly related to noise bandwidth, i.e., the low or high cut-off frequencies and can be uniquely determined from the correlation, $\rho _1$, between adjacent ISIs (Eqs. 20 and 24). More robust estimates are obtained from the ratio $\rho _2/\rho _1$. SCCs at subsequent lags are related to *a* as terms in a geometric progression (Eqs. 19 and 23).
6. While more complex patterns of SCCs can be produced by other types of noise correlation functions, only Type I and Type II SCC patterns are observed in P-type afferent spike-trains. Type III SCC patterns are mentioned here because they are commonly reported in modeling studies and are discussed further below.
7. The expression for ISI SCCs is independent of the adaptive threshold parameters (*A* and $\tau$), the signal (*v*), and the firing threshold ($\gamma$). It is dependent only on the noise correlation function, including noise power ${{\,\mathrm{\mathbf {R}}\,}}_0$.
8. The model fits ISI and joint-ISI distributions.
9. For both non-bursting and bursting units (slow and fast noise, respectively) the theoretical prediction of the sum of ISI SCCs is exactly $-0.5$. The sum of SCCs over all afferent spike trains is close to this limit: $-0.475 \pm 0.04.$ ($N = 52$).
10. SNR ($10\,\log _{10}(v^2/{{\,\mathrm{\mathbf {R}}\,}}_0$) is generally larger than $20~\text {dB}$, i.e., fluctuations in threshold (noise) are small compared to the input signal. This is in keeping with the hypothesis that spike-time jitter is small in comparison with the mean ISI.

There are two components to ISI serial correlations as is apparent from Eq. (9), where the *i*th ISI is given by $t_{i} - t_{i-1} = \left( \gamma _{i} - \gamma _{i-1} + A\right) /m$. The first component is due to the difference $\gamma _{i} - \gamma _{i-1}$ which is coupled to the next (adjacent) interval $t_{i+1} - t_{i}$ by the common term $\gamma _i$. This term appears with opposing signs in adjacent ISIs and hence results in a negative correlation which does not extend beyond these ISIs. If the $\gamma _i$ are uncorrelated then it can be shown that the adjacent ISI correlation $\rho _1 = -0.5$ and all other $\rho _k = 0$, for $k \ge 2$. Thus, for independent random variables, this result follows from the property of a differencing operation and it is not indicative of memory beyond adjacent ISIs. The second component of ISI correlations is due to long-range correlations ${{\,\mathrm{\mathbf {R}}\,}}$ in the random process $\gamma$ which extend beyond adjacent ISIs. These correlations are endogenous, possibly biophysical in origin, and could be shaped by coding requirements. The two components to ISI correlations are made clear by restating Eq. (13) for $\rho _1$ as

29 $$\begin{aligned} \rho _1 = -\frac{1}{2} + \frac{{{\,\mathrm{\mathbf {R}}\,}}_1-{{\,\mathrm{\mathbf {R}}\,}}_2}{2\left( {{\,\mathrm{\mathbf {R}}\,}}_0-{{\,\mathrm{\mathbf {R}}\,}}_1\right) }\,. \end{aligned}$$

Thus, they are separable. For a wide-sense stationary process, ${{\,\mathrm{\mathbf {R}}\,}}_0 > {{\,\mathrm{\mathbf {R}}\,}}_k$ for all $k \ge 1$, and so the denominator of the second term is always positive. Thus, the deviation of $\rho _1$ from $-0.5$ is determined by the sign of ${{\,\mathrm{\mathbf {R}}\,}}_1-{{\,\mathrm{\mathbf {R}}\,}}_2$. This term is positive for non-bursting units (Type I SCCs), and it is negative for bursting units (Type II SCCs). The singleton case (Type III SCCs) results because noise is uncorrelated and so the term vanishes. In this case only the first component is present. For a Ornstein–Uhlenbeck or Gauss–Markov process, i.e., first-order AR process with coefficient $a > 0$, ${{\,\mathrm{\mathbf {R}}\,}}_1 > {{\,\mathrm{\mathbf {R}}\,}}_2$ (Fig. 5A), and this produces a non-bursting Type I pattern. When the AR parameter is negative, i.e., the coefficient is $-a$, ${{\,\mathrm{\mathbf {R}}\,}}_1 < {{\,\mathrm{\mathbf {R}}\,}}_2$ (Fig. 5B, C), and this produces a bursting Type II pattern with a bimodal ISI distribution. Thus, a single parameter (the sign of the first-order AR parameter) can create the observed patterns of negatively correlated SCCs. Type III SCCs where $\rho _1 = -0.5$, and $\rho _k = 0$ for $k \ge 2$ are trivially generated by perturbing the spike threshold with uncorrelated white noise, i.e., by setting $a = 0$ in the first-order AR process. We have not observed Type III neurons experimentally although some spike trains have $\rho _1$ values close to $-0.5$.

The sign of the *a* parameter in the AR process determines the time-scale of noise fluctuations in the spike threshold, and determines the patterns of SCCs. Recall that $\tau _\gamma = -T_1/\ln \left( a\right)$. For Type I SCCs, the AR process produces slow noise with noise bandwidth dominated by frequencies $\omega < 2\pi \tau _\gamma ^{-1}$ (i.e., low-pass). For Type II SCCs, the generating noise is dominated by frequencies $\omega > 2\pi \tau _\gamma ^{-1}$ (i.e., high-pass). In the latter case, the characteristic ringing of the correlation function is distinctive, with the degree of damping being controlled by the value of $\tau _\gamma$.

From the above observations, we suggest that the ARMA process leading to generation of ISIs is the result of two processes: 1) An MA component which is due to a differencing operation. This contributes a value of $-1/2$ to $\rho _1$, i.e., to the finite portion of the autocorrelation function (and the infinite tail of the partial autocorrelation function). 2) An AR component which is due to a feedback loop (Fig. 4). This contributes a value of $-1/2$ to $\phi _{1,\,1}$, i.e., to the finite portion of the partial autocorrelation function (and the infinite tail of the autocorrelation function). In the noise process used here, $\rho _1 = -1/2 \pm a/2$ (Eqs. 20 and 24 for non-bursting and bursting afferents, respectively), and so the residual contribution of the autoregressive component to $\rho _1$ is $\pm a/2$. The goodness of fit to the experimental data with a single parameter $\pm a$ leads us to believe that the underlying ARMA model is first order in both the AR and MA components.

Farkhooi et al. (2009) reported that in the extrinsic neurons of the mushroom body in the honeybee, the ISI partial autocorrelation function is zero for all lags $k > 1$. Thus, the ISI process is first-order AR, i.e., AR(1). As we remarked above, we cannot conclude the order of the AR or ARMA process from our data although we think that it is possibly ARMA$(1,\,1)$. The threshold noise process (whose autocorrelation and partial autocorrelation are depicted in Fig. 5) is first-order AR (Figs. 5D–F). However, the threshold noise correlations (Eq. 18 for Type I, and Eq. 22 for Type II) influences the ISI correlations according to Eq. 13. This is illustrated in the closed form expressions for ISI SCCs using a first-order noise process (Eqs. 19 and 23). The ISI SCC $\rho _k$ is a function of *k* and $k-1$. For a first-order noise process at least, our data suggest that the ISI process is a mix of AR and MA processes and not a simple AR(1) process.

### Comparison with other adaptation models

These results can be directly compared with results from an earlier study which modeled adaptation currents (Schwalger et al. 2010). In that study, patterns of SCCs were generated by a perfect integrate-and-fire neuron under two conditions : i) a deterministic adaptation current with fast, white Gaussian noise input, and ii) a stochastic adaptation current with slow, exponentially correlated (channel) noise. A deterministic adaptation current with fast noise produced patterns of SCCs that we report here as Types I and II. These patterns were characterized by a parameter $\vartheta$ which is analogous to the AR parameter *a* used here, with Type I pattern (positive coefficient, *a*) corresponding to $\vartheta >0$, and Type II pattern (negative coefficient, $-a$) corresponding to $\vartheta < 0$. A pattern similar to the Type III SCC pattern reported here resulted when $\vartheta = 0$. This is the same as $a = 0$ although we did not observe these neurons experimentally. While we define Type III SCCs to have only one pattern ($\rho _1 = -0.5$, and $\rho _k = 0$ for $k \ge 2$), Schwalger et al. (2010) report that $\rho _k = 0$ when $k \ge 2$, but the value of $\rho _1$ is governed by an additional parameter and could take a range of values, with $\rho _1 = -0.5$ appearing as a limiting case. Stochastic adaptation currents produced only positive ISI correlations which we do not observe or model here. In a subsequent report Schwalger and Lindner (2013) extended the model to more general integrate-and-fire models and obtained a relationship between ISI SCCs and the phase-response curve (PRC). The patterns of SCCs reported here most likely correspond to their Type I PRC.

The model presented here for first-order noise processes, given by Eqs. (17) and (21), does not generate positive correlations. Note that the parameter *a* in these equations is bounded such that $0< a = \exp (-T_1/\tau _\gamma ) < 1$. Thus, the AR(1) coefficients *a* in Eq. (17) and $-a$ in Eq. (21), are restricted to the open interval $(-1,\,1)$ otherwise the noise process is unstable (i.e., unbounded). Let us say that we require the first ISI SCC $\rho _1 > 0$, then from Eq. (19) we must have $a > 1$ (Type I neuron), and from Eq. (23) we must have $-a < -1$ (Type II neuron). Thus, *a* is outside the stable range of the AR(1) coefficients and the model cannot generate positive ISI SCCs. For second-order or higher-order noise processes $\text {AR}(p)$ with $p > 1$, positive ISI SCCs are possible if the noise correlation ${{\,\mathrm{\mathbf {R}}\,}}_k$ satisfies ${{\,\mathrm{\mathbf {R}}\,}}_0 < 2{{\,\mathrm{\mathbf {R}}\,}}_1 - {{\,\mathrm{\mathbf {R}}\,}}_2$ (from Eq. 13). It should be possible to construct arbitrary correlation sequences using Eq. (13) and satisfying the inequality, to create prescribed sequences of ISI correlations. We have not explored these ideas.

The SCCs reported here follow a geometric progression with the filter-pole *a* being the ratio parameter (see Eqs. 19 and 23). Schwalger et al. (2010) and Schwalger and Lindner (2013) reported that the patterns of negative correlations follow a geometric progression with the ratio parameter being $\vartheta$. Similarly, a geometric progression was also reported by Urdapilleta (2011). Note that a first-order Markov process also follows a geometric progression with ratio parameter $\rho _1$ (Cox and Lewis 1966; Nakahama et al. 1972). A geometric progression of SCCs is not surprising given that the ISI sequence is discrete, and thus, feedback will result in a (discrete) recurrence relation. For example, all the first-order AR processes used to predict SCCs have correlation functions which are in geometric progression (Eqs. 19, 22). Taken together with the analysis of partial correlation coefficients, and as noted above, it is likely that the underlying ARMA process generating the ISI sequence is low-order. The limiting sum of SCCs reported here (Eq. 14) is exactly $-0.5$, whereas Schwalger and Lindner (2013) report a sum that asymptotically approaches a value that is slightly larger than $-0.5$. The average sum of SCCs in our experimental data (Fig. 2D) is also slightly larger than $-0.5$ ($-0.475 \pm 0.04.$), and this merits further investigation.

In considering the results presented here using a dynamical threshold model, and those from the more general models using an adaptation current, it appears that the presence of the feedback loop (the coupling between the membrane voltage and the dynamic threshold, see Figs. 3B, 4B, and 10B) may account for almost all the properties of SCCs if the noise fluctuation is shaped appropriately. These fluctuations have the effect of reverberating around the feedback loop, creating memory and introducing negative correlations extending over multiple ISIs. However, the source of this noise is moot because it can appear in the input or the model parameters (see Eq. 31, where noise can be distributed over the input *v*, the threshold decay function *r*, or the spike threshold $\gamma$). The dynamic threshold model used here does not suggest a biophysical mechanism, but we suggest that a noisy threshold is due to an endogenous source of noise which perturbs the spiking threshold $\gamma$ or the time-constant $\tau$, i.e., noise is not passed through the input. This is an assumption, and input noise will of course shape SCCs. However, several biophysical mechanisms can account for endogenous sources of noise, including probabilistic transitions between conformational states in voltage-gated ion channels (White et al. 1998, 2000; Schneidman et al. 1998; Van Rossum et al. 2003; Fontaine et al. 2014) leading to perturbations in gating characteristics (Benda et al. 2010; Chacron et al. 2007). These can introduce cumulative refractoriness in firing. It should be noted that the amount of noise to be added to the threshold is small, generally weaker than $20~\text {dB}$ SNR (see Figs. 6, 7, and 8). That only weak noise may be necessary has been reported earlier by Schwalger and Lindner (2013), and in a study on threshold shifts by Fontaine et al. (2014).

While a dynamic threshold model does not explicitly incorporate biophysics, models with adaptation currents can be more readily tied to biophysical conductances. An ion channel that may be a substantial contributor to adaptation currents is the non-inactivating M-current (KCNQ/Kv7 family, Brown and Adams 1980) which is a voltage-gated potassium channel with slow dynamics (relative to the mean ISI). This channel may be responsible for spike-timing precision, and hence a timing-based neural code (see Jones et al. 2015, for a discussion). The possible role of the M-current and the specific sources of noise have been explored earlier (see Schwalger et al. (2010) above, in relation to types of SCCs, and Fisch et al. (2012)). Both studies (Schwalger et al. 2010; Fisch et al. 2012) included a modified conductance-based Traub-Miles model (Traub and Miles 1991; Ermentrout 1998) frequently used in modeling M-currents. They concluded that negative correlations were a consequence of a deterministic adaptation current with fast white Gaussian noise input rather than a stochastic adaptation current with slow correlated noise. The current report shows that at least some of the results with adaptation currents can be reproduced with a simple dynamic threshold with a noisy spiking threshold or a noisy decay time-constant. Further, either slow noise or fast noise can produce negative correlations if injected into the spiking threshold, with the time-scale of noise fluctuations determining the type of SCC pattern that is produced (i.e., bursting or non-bursting). In another study (Benda et al. 2010) the first SCC ($\rho _1$) was compared when the generating models were a leaky integrate-and-fire (LIF) neuron with an adaptation current (LIFAC) or a dynamic threshold (LIFDT). When the LIFAC and LIFDT models are matched so that the spike-rate and adaptation are about the same, the $\rho _1$ as a function of spike rate are also similar. This gives reason to believe that either model of adaptation can give rise to similar patterns of SCCs, possibly due to the simple feedback present in the models. However, a more detailed biophysical investigation is needed before we can tease apart the differences at a mechanistic level.

Finally, we note that the model has been formulated as a dynamic threshold model. However, the model is so minimal that it can be viewed in some instances as an adaptation current. For example, when we transformed the randomness in spike threshold $\gamma$ into a variable time-constant (across ISIs), we have a model which has a constant spike threshold but a variable relaxation (outward) current. This transformed model is more akin to an adaptation current although it is not stochastic (it is deterministic within an ISI). Thus, for a simple, abstract model such as the one presented here, it may not be possible to make a distinction between dynamic threshold and adaptation current.

### Power spectra and noise-shaping

Negatively correlated spike trains have implications for information transmission (Chacron et al. 2004b). These spike trains have a power spectra $P\left( \omega \right)$ that rolls off toward DC (Chacron et al. 2004a; Schwalger and Lindner 2013), effectively reducing low-frequency noise and improving SNR in the baseband (also referred to as noise-shaping). The connection between DC power $P\left( \omega = 0\right)$ and the ISI SCCs is given by Eq. (16) (Cox and Lewis 1966), where it can be seen that negative correlations have a tendency to reduce noise at zero-frequency. From the theoretical limit of the sum of SCCs ($-0.5$) reported here in Eq. (14), similar to the results with adaptation currents (Schwalger and Lindner 2013), DC-power vanishes, thus yielding a perfect DC block allowing low-frequency signals to be transmitted with very high SNR. The limiting sum of observed SCCs is only just a little larger than the optimum sum ($-0.5$), and this allows some noise to bleed through at zero-frequency. These results are in line with the results on power spectra first predicted by Chacron et al. (2004b) and Chacron et al. (2004a), and more recently extended to models with adaptation currents (Schwalger and Lindner 2013).

### Implications for optimal coding

The model presented here sets the spike threshold $\gamma = A/2$. We showed previously that the time-varying dynamic threshold $r\left( t\right)$ is an estimator of the input signal *v*(*t*), and the mean-squared estimation error $\langle \left( v(t) - r(t)\right) ^2\rangle$ is minimized when the firing threshold is $\gamma = A/2$ (Jones et al. 2015; Johnson et al. 2015). The error increases for any other value of $\gamma$, in particular $\gamma = 0$ which is the value usually adopted in the literature. Thus, for optimal timing of spikes the ongoing estimation error must be bounded below by $\gamma = A/2$. When this bound is reached, the neuron fires a spike and raises the adaptive threshold variable by *A*. Viewed in this manner, the spike generator directly encodes the error and not the signal (Figs. 3B, 4B). This is much more efficient than directly encoding the signal (or some filtered version of the signal). The coding error is equivalent to quantization error in digital coding. This coding mechanism is analogous to one-bit delta modulation (Jayant and Noll 1984), but it is asymmetric because the threshold is one-sided. Thus, coding with an dynamic threshold is analogous to lossy digital coding or source coding. That is, a neuron performs data compression.

The optimal coding principle is based on a trade-off between maximizing coding fidelity (reducing estimation error) and minimizing the long-term firing rate of the neuron (a metabolic energy constraint, see Attwell and Laughlin (2001), and Johnson et al. (2016); Jones et al. (2015); Johnson et al. (2015)). This formulation is closely related to an approach from predictive coding in spiking networks which also balances fidelity against spiking activity in a population of spiking neurons (Boerlin et al. 2013).

For a constant DC-valued input, the SCCs generated by the model are dependent solely on noise correlations ${{\,\mathrm{\mathbf {R}}\,}}$, and are independent of all other parameters, including the dynamic threshold parameters *A* and $\tau$, the mean value of the threshold $\gamma$, and the constant DC-valued input signal *v*. The model parameters *A* and $\tau$ are fitted from the baseline (spontaneous) spike-train through the relationship $A\tau = vT_1$, where *v* is the bias input and $T_1$ is the long-term mean ISI. This allows us to reduce the degrees of freedom to one, to either *A* or $\tau$, and then determine the free parameter by optimizing the model spike train to match experimental spike-times (see Jones et al. 2015, for details of the procedure). Thus, the dynamic threshold parameters are not estimated from the observed SCCs. This fits with our understanding of the dynamic threshold as an optimal estimator with its parameters being determined by coding quality and an energy constraint (Jones et al. 2015; Johnson et al. 2015). When the deterministic parameters are fixed, a noisy threshold can be generated by estimating the filter parameter *a* from $\rho _1$ or $\rho _2/\rho _1$ to match the observed SCCs. This decoupling of dynamic threshold parameters from SCCs implies that a correlation function ${{\,\mathrm{\mathbf {R}}\,}}$ can be generated from a specified sequence of SCCs (determined, say experimentally) without reference to the time-varying threshold. Thus, a family of spike trains with arbitrary mean ISI, ISI and joint-ISI distributions can be generated, all of which have the same sequence or pattern of SCCs. Some iteration will be needed to fit the ISI and joint-ISI distributions from the dynamic threshold parameters *A* and $\tau$ as is done here, but this is not too difficult.

### Further work and conclusion

The main goal of this work was to show that a simple dynamic threshold model with few parameters can reproduce known negative correlations, and further, model experimental data and provide insight into the origin and patterns of ISI negative correlations. We proceeded on the assumption that there is only one experimentally observable quantity: the sequence of spike times. Based on spike-times, we have shown that the ISI distribution, the joint-ISI distribution, and SCCs can be predicted accurately. The approach used here suffers from several drawbacks. The mean firing rate, or equivalently mean ISI, is implicitly defined through the model parameters and hence it does not influence SCCs (the noise correlation function is discretized using ISI lag-number, and not the absolute value of the ISI). The SCCs obtained with this approach are thus invariant under firing rate. In reality, we expect correlations to be small when the spike rate is high or low, reaching a maximum at some intermediate firing rate (see Benda et al. 2010). The independence of SCCs from mean ISI is a result of infinite memory in the model because the exponential threshold was approximated as a line with slope *m*. A second drawback is that the input is a fixed DC-value and does not incorporate dynamics or noise. It should be noted that noise in the current model is assumed to perturb the spiking threshold; however, it can be exogenous, i.e., present in the input *v*, or present jointly in $\gamma$ and *v*. Currently, there is no way to distinguish between these cases (see the deterministic firing rule specified in Eq. 7) because noise can be distributed over $\gamma$ or over *v*. Thus, for a noisy DC-valued input and fixed $\gamma$, the results will be the same as for a noisy $\gamma$. Rather than determining the effect of input dynamics on SCCs an alternative and promising line of enquiry is to determine the role of ISI SCCs, (determined under spontaneously active conditions) on processing time-varying inputs of varying bandwidth, i.e., on signal encoding. As noted earlier (Jones et al. 2015), the bandwidth of the input will affect the quality of the coding and the extent to which ISI correlations maintain coding fidelity (i.e., the stimulus estimation error). To determine these effects, the model can be readily adapted to non-constant inputs by making the input piece-wise linear between spikes. This approach and the necessary theory was developed earlier with a deterministic version of the dynamic threshold model to predict spike-times (Jones et al. 2015; Johnson et al. 2015, 2016). We hypothesize that a stochastic extension of the model with weak noise (added to the spike threshold or decay time-constant) should not disrupt timing other than introduce timing jitter. This has been shown in neocortical neurons where spike-timing is reliable across repeated presentations of rapidly fluctuating time-varying signals (Mainen and Sejnowski 1995), and can be accurately modeled using a dynamic threshold (Jones et al. 2015). Another study, albeit preliminary, showed that the patterns of negative correlations do not change when there is a time varying input (Ratnam et al. 2001). While it is known that negative correlations, and more generally adaptation currents can reduce variability and stabilize firing rate in single neurons (see for example, Ratnam and Nelson 2000; Schwalger and Lindner 2013) and populations of neurons (Farkhooi et al. 2011, 2013), robust spike-times (i.e., reduced timing jitter) in response to time-varying signals may be yet another benefit provided by negative SCCs in ISIs. Thus, the use of a more exact, i.e., leaky, dynamic threshold function, the extension to time-varying inputs, and the determination of the linkages between dynamics of the input, the threshold, and coding fidelity, are the obvious next steps.

The simplified dynamic threshold model used here demonstrates the influence of noise on ISI correlations. We provide a closed-form expression for SCCs as a function of the noise correlation ${{\,\mathrm{\mathbf {R}}\,}}$. This is useful for solving the inverse problem when SCCs are known and the discrete sequence ${{\,\mathrm{\mathbf {R}}\,}}_k$ is to be determined. The forward problem is also readily solved, i.e., given a sequence ${{\,\mathrm{\mathbf {R}}\,}}_k$, the SCCs can be evaluated. We illustrate with a first-order AR model, and show that a single-parameter (the sign of the coefficient in the first-order AR process) captures the pattern of observed SCCs, and in this respect it agrees with a detailed dynamical model using adaptation currents (Schwalger and Lindner 2013). This model can fit observed spike-times with good accuracy (Kobayashi et al. 2009; Jones et al. 2015), is energy-efficient while maximizing coding fidelity (Jones et al. 2015; Johnson et al. 2015), and is computationally inexpensive. Finally, the model provides a quick and efficient way to generate surrogate data mimicking negatively correlated spike trains observed in experimental neurons. Thus, the model can be useful for understanding timing-based neural codes.

## Appendix

### Deterministic and stochastic adaptive threshold model

In the deterministic adaptive threshold model (Fig. 3A), if $t_{i-1}$ and $t_{i}$, $i \ge 1$, are successive spike-times, then the time evolution of the adaptive threshold $r\left( t\right)$ is given by

30 $$\begin{aligned} r\left( t\right)= & {} (v - \gamma + A)\,\exp \left( -\frac{t - t_{i-1}}{\tau }\right) ,\quad t_{i-1} < t \le t_{i}, \nonumber \\= & {} (v - \gamma + A)\left( 1 - \frac{t - t_{i-1}}{\tau }\right) + {{\,\mathrm{\mathrm {O}}\,}}(\left( t - t_{i-1}\right) ^2). \end{aligned}$$

At $t = t_{i-1}^+$, $r\left( t_{i-1}^+\right) = v - \gamma + A$, and at $t = t_{i}$, $r\left( t_{i}\right) = v - \gamma$. So,

31 $$\begin{aligned} r\left( t\right) - r\left( t_{i-1}\right) = -\frac{v - \gamma + A}{\tau }(t - t_{i-1}),\quad t_{i-1} < t \le t_{i}. \end{aligned}$$

Let $m = \left( v -\gamma + A\right) /\tau > 0$, so that the slope of the adapting threshold is $-m$, then

32 $$\begin{aligned} r\left( t\right) - r\left( t_{i-1}\right) = -m(t - t_{i-1}),\quad t_{i-1} < t \le t_{i}. \end{aligned}$$

Noting that $$r\left( t_{i}\right) = v - \gamma $$,

33 $$\begin{aligned} \frac{A}{m} = \left( t_{i} - t_{i-1}\right) , \end{aligned}$$

which is the deterministic firing threshold for a constant, DC-level signal.

#### Dynamic threshold with random firing threshold

Throughout this work, we will use the term covariance to refer to the mean subtracted correlation. If the two random variables in question are identical, then we will simply refer to covariance as variance, and correlation as autocorrelation. If the correlation is normalized by the variance, then we will refer to it as the correlation coefficient.

We will now provide a stochastic extension to the model by making the spike threshold ($\gamma$) noisy while leaving all else unchanged (Fig. 4). Let $\gamma$ be a discrete wide-sense stationary process with mean ${{\,\mathrm{\mathbf {E}}\,}}[\gamma ]$, discrete autocorrelation function ${{\,\mathrm{\mathbf {R}}\,}}_k$ and power ${{\,\mathrm{\mathbf {E}}\,}}\left[ \gamma ^2\right] = {{\,\mathrm{\mathbf {R}}\,}}_0$. The spike threshold with additive noise assumes the value $\gamma _i$, $i \ge 1$ immediately after the $(i-1)$th spike and remains constant in the time interval $t_{i-1} < t \le t_{i}$ (Fig. 4A) (Chacron et al. 2004a; Gestri et al. 1980). Thus, the *i*th spike is emitted when the error satisfies the condition

34 $$\begin{aligned} e\left( t_i\right) \ge \gamma _i, \quad \text {for} \, t > t_{i-1}. \end{aligned}$$

Subsequently the adaptive threshold jumps to a higher value specified by $v - \gamma _i + A$, and the noisy spike threshold assumes a new value $\gamma _{i+1}$. From Fig. 4A, proceeding as before

35 $$\begin{aligned} t_{i} - t_{i-1} = \frac{1}{m}\left\{ \gamma _{i} - \gamma _{i-1} + A\right\} . \end{aligned}$$

The mean ISI is therefore ${{\,\mathrm{\mathbf {E}}\,}}\left[ t_{i}-t_{i-1}\right] = A/m$ as in the deterministic case given by Eq. (7). Thus, the correlation function for the ISI sequence is

36 $$\begin{aligned} {{\,\mathrm{\mathbf {E}}\,}}[\left( t_i-t_{i-1}\right) ^2]= & {} \frac{1}{m^2}{{\,\mathrm{\mathbf {E}}\,}}\left[ \left( \gamma _{i} - \gamma _{i-1} + A\right) \left( \gamma _{i} - \gamma _{i-1} + A\right) \right] , \nonumber \\= & {} \frac{A^2}{m^2} - \frac{2}{m^2}({{\,\mathrm{\mathbf {E}}\,}}[\gamma _i\, \gamma _{i-1}] - {{\,\mathrm{\mathbf {E}}\,}}[\gamma ^2])\,, \end{aligned}$$

with variance

37 $$\begin{aligned} {{\,\mathrm{\mathrm {Var}}\,}}\left( t_i-t_{i-1}\right) = -\frac{2}{m^2}\left( {{\,\mathrm{\mathbf {E}}\,}}\left[ \gamma _{i}\,\gamma _{i-1}\right] - {{\,\mathrm{\mathbf {E}}\,}}\left[ \gamma ^2\right] \right) . \end{aligned}$$

More generally, for the sum of *k* ISIs

38 $$\begin{aligned} {{\,\mathrm{\mathbf {E}}\,}}[\left( t_{i+k}-t_{i}\right) ^2]= & {} \frac{1}{m^2}{{\,\mathrm{\mathbf {E}}\,}}[\left( \gamma _{i+k} - \gamma _{i} + A\right) ^2], \nonumber \\= & {} \frac{kA^2}{m^2} - \frac{2}{m^2}({{\,\mathrm{\mathbf {E}}\,}}[\gamma _{i+k}\,\gamma _i] - {{\,\mathrm{\mathbf {E}}\,}}[\gamma ^2])\,, \end{aligned}$$

with variance

39 $$\begin{aligned} {{\,\mathrm{\mathrm {Var}}\,}}\left( t_{i+k}-t_{i}\right) = -\frac{2}{m^2}({{\,\mathrm{\mathbf {E}}\,}}\left[ \gamma _{i+k}\,\gamma _{i}\right] - {{\,\mathrm{\mathbf {E}}\,}}\left[ \gamma ^2\right] ). \end{aligned}$$

From Eqs. (36) and (38), it can be seen that there are two terms in each of these ISI correlation functions. The first is due to the input signal (which gives a mean ISI of *A*/*m*), and the second is due to the discrete-event noise process $\gamma$. From the assumption of wide-sense stationarity, the autocorrelation function ${{\,\mathrm{\mathbf {E}}\,}}\left[ \gamma _i\, \gamma _j\right]$ can be written as ${{\,\mathrm{\mathbf {R}}\,}}\left( t_i - t_j\right)$. The discrete nature of the correlation function ${{\,\mathrm{\mathbf {R}}\,}}$ is made clear in the following way. Denote the mean of the *k*th-order interval by $T_k = {{\,\mathrm{\mathbf {E}}\,}}[t_{i+k} - t_i]$, then the mean ISI is $T_1$ $(=A/m)$, and further $T_k = kT_1$. The random variable $\gamma$ is generated once every ISI and thus, the discrete autocorrelation function ${{\,\mathrm{\mathbf {R}}\,}}$ takes values at successive multiples of the mean ISI, i.e., ${{\,\mathrm{\mathbf {R}}\,}}\left( T_1\right)$, ${{\,\mathrm{\mathbf {R}}\,}}\left( T_2\right)$, etc., and will be denoted by ${{\,\mathrm{\mathbf {R}}\,}}_1$, ${{\,\mathrm{\mathbf {R}}\,}}_2$, etc., respectively. As noted before, ${{\,\mathrm{\mathbf {R}}\,}}_0$ is noise power. That is, we can write

40 $$\begin{aligned} {{\,\mathrm{\mathbf {R}}\,}}\left( t_{i+k} - t_i\right) = {{\,\mathrm{\mathbf {R}}\,}}\left( T_k\right) = {{\,\mathrm{\mathbf {R}}\,}}_k\,, \end{aligned}$$

and therefore

41 $$\begin{aligned} {{\,\mathrm{\mathrm {Var}}\,}}\left( t_{i}-t_{i-1}\right)= & {} \frac{2}{m^2}\left\{ {{\,\mathrm{\mathbf {R}}\,}}_0 - {{\,\mathrm{\mathbf {R}}\,}}_1\right\} \,, \end{aligned}$$

42 $$\begin{aligned} {{\,\mathrm{\mathrm {Var}}\,}}\left( t_{i+k}-t_{i}\right)= & {} \frac{2}{m^2}\left\{ {{\,\mathrm{\mathbf {R}}\,}}_0 - {{\,\mathrm{\mathbf {R}}\,}}_k\right\} \,. \end{aligned}$$

The covariance of two ISIs $\left( t_i-t_{i-1}\right)$ and $\left( t_{i+k}-t_{i+k-1}\right)$ separated by lag $k \ge 1$ is

43 $$\begin{aligned}&{{\,\mathrm{\mathrm {Cov}}\,}}\left( \left( t_i-t_{i-1}\right) ,\left( t_{i+k}-t_{i+k-1}\right) \right) \nonumber \\&\quad = \frac{1}{m^2}{{\,\mathrm{\mathbf {E}}\,}}[\left( \left( \gamma _i-\gamma _{i-1}\right) + A\right) \left( \left( \gamma _{i+k}-\gamma _{i+k-1}\right) +A\right) ] - \frac{A^2}{m^2}\,, \nonumber \\&\qquad \frac{1}{m^2}{{\,\mathrm{\mathbf {E}}\,}}[\gamma _i\,\gamma _{i+k} - \gamma _i\,\gamma _{i+k-1} + \gamma _i\,A - \gamma _{i-1}\,\gamma _{i+k} \nonumber \\&\qquad + \gamma _{i-1}\,\gamma _{i+k-1} - \gamma _{i-1}\,A +\gamma _{i+k}\,A - \gamma _{i+k-1}\,A \nonumber \\&\qquad + A^2] - \frac{A^2}{m^2}\,. \end{aligned}$$

This yields

44 $$\begin{aligned}&{{\,\mathrm{\mathrm {Cov}}\,}}\left( \left( t_i-t_{i-1}\right) ,\left( t_{i+k}-t_{i+k-1}\right) \right) \nonumber \\&\quad = \frac{1}{m^2}{{\,\mathrm{\mathbf {E}}\,}}[\gamma _i\,\gamma _{i+k} - \gamma _i\,\gamma _{i+k-1} - \gamma _{i-1}\,\gamma _{i+k} + \gamma _{i-1}\,\gamma _{i+k-1}]\,, \nonumber \\&\quad = -\frac{1}{m^2}\left\{ {{\,\mathrm{\mathbf {R}}\,}}_{k-1} -2{{\,\mathrm{\mathbf {R}}\,}}_k + {{\,\mathrm{\mathbf {R}}\,}}_{k+1}\right\} \,. \end{aligned}$$

The definition of serial correlation coefficient at lag *k* is (Cox and Lewis 1966),

45 $$\begin{aligned} \rho _k = \frac{{{\,\mathrm{\mathrm {Cov}}\,}}\left( \left( t_{i}-t_{i-1}\right) ,\,\left( t_{i+k}-t_{i+k-1}\right) \right) }{{{\,\mathrm{\mathrm {Var}}\,}}\left( t_{i}-t_{i-1}\right) ^{1/2}{{\,\mathrm{\mathrm {Var}}\,}}\left( t_{i+k}-t_{i+k-1}\right) ^{1/2}}\,. \end{aligned}$$

For a wide-sense stationary process, the covariances are constant, and the subscript *i* can be dropped. Introducing Eqs. (41) and (44) into Eq. (45) yields

46 $$\begin{aligned} \rho _0= & {} 1, \end{aligned}$$

47 $$\begin{aligned} \rho _k= & {} -\frac{{{\,\mathrm{\mathbf {R}}\,}}_{k-1} - 2{{\,\mathrm{\mathbf {R}}\,}}_{k} + {{\,\mathrm{\mathbf {R}}\,}}_{k+1}}{2\left( {{\,\mathrm{\mathbf {R}}\,}}_0-{{\,\mathrm{\mathbf {R}}\,}}_1\right) }\,, \quad \ k \ge 1\,. \end{aligned}$$

These equations are reproduced in Results as Eqs. (12) and (13), respectively. The serial-correlation coefficients (SCCs) given by Eqs. (46) and (47) are independent of the slope *m* of the reconstruction filter (the decay rate of the adaptive threshold) and the reconstruction filter gain *A*. Thus, the observed correlation structure of the spike-train is determined solely by the noise statistics of the firing threshold $\gamma$.

#### Dynamic threshold with a random time-constant

We begin with Eqs. (9), (25), and (26), which transform a random firing threshold into a random time-constant $$\tau _i$$ that is held constant between spike-times $$[t_{i-1}, t_{i})$$ with mean $$\tau = {{\,\mathrm{\mathbf {E}}\,}}\left[ \tau _i\right] $$ (Fig. 10). It is immediately apparent that the covariance of the sequence $$\tau _i$$ (sampled at the ISIs) is the same as the covariance of the ISI sequence up to a scale-factor, and therefore the serial correlations of $$\tau $$ ($$\rho _{\tau ,\,k}$$, $$k \ge 0$$) are the same as the serial correlations of the ISIs. We can establish this as follows. From Eq. (26)

48 $$\begin{aligned} {{\,\mathrm{\mathrm {Var}}\,}}\left( \tau _i\right) = \frac{2\tau ^2}{A^2}\left\{ {{\,\mathrm{\mathbf {R}}\,}}_0 - {{\,\mathrm{\mathbf {R}}\,}}_1\right\} \,, \end{aligned}$$

and

49 $$\begin{aligned} {{\,\mathrm{\mathrm {Cov}}\,}}\left( \tau _{i+k}\,,\tau _{i}\right) = -\frac{\tau ^2}{A^2}\left\{ {{\,\mathrm{\mathbf {R}}\,}}_{k-1} - 2{{\,\mathrm{\mathbf {R}}\,}}_k + {{\,\mathrm{\mathbf {R}}\,}}_{k+1}\right\} \,. \quad k \ge 1. \end{aligned}$$

From Eqs. (45), (48) and (49), we can determine the serial correlation coefficients of the random time-constants to be

50 $$\begin{aligned} \rho _{\tau ,\,0}= & {} 1, \end{aligned}$$

51 $$\begin{aligned} \rho _{\tau ,\,k}= & {} -\frac{{{\,\mathrm{\mathbf {R}}\,}}_{k-1} - 2{{\,\mathrm{\mathbf {R}}\,}}_k + {{\,\mathrm{\mathbf {R}}\,}}_{k+1}}{2\left( {{\,\mathrm{\mathbf {R}}\,}}_0-{{\,\mathrm{\mathbf {R}}\,}}_1\right) }, \quad \ k \ge 1. \end{aligned}$$

These equations are reproduced in Results as Eqs. (27) and (28), respectively. The right side of the above equations, Eqs. (50) and (51), are the same as Eqs. (46) and (47), which are the expressions for the SCCs of a spike train generated with a noisy adaptive threshold. Thus, the random filter time-constants have the same serial correlation coefficients as the interspike intervals, $\rho _{\tau ,\,k} = \rho _k$. This is a “pass through” effect where the correlations observed in the time-constant are directly reflected in the ISI correlations.

In summary, a noisy threshold or a noisy dynamic threshold time-constant can generate spike trains with the same ISIs and SCCs, and either method can be used.

### Sum of serial correlation coefficients

We now determine the limiting sum of the SCCs over all lags. Let us first transform $\rho _k$ as follows by defining

52 $$\begin{aligned} z_k = -2\rho _k\left( {{\,\mathrm{\mathbf {R}}\,}}_0-{{\,\mathrm{\mathbf {R}}\,}}_1\right) \,, \quad k \ge 1\,. \end{aligned}$$

Then, from Eq. (47), $z_k$ is a moving-average process such that

53 $$\begin{aligned} z_k = {{\,\mathrm{\mathbf {R}}\,}}_{k-1} - 2{{\,\mathrm{\mathbf {R}}\,}}_k + {{\,\mathrm{\mathbf {R}}\,}}_{k+1}\,, \end{aligned}$$

from which it is easy to show that

54 $$\begin{aligned} \sum _{k=1}^{N} z_k = {{\,\mathrm{\mathbf {R}}\,}}_{N+1} - {{\,\mathrm{\mathbf {R}}\,}}_N + {{\,\mathrm{\mathbf {R}}\,}}_0 - {{\,\mathrm{\mathbf {R}}\,}}_1\,, \end{aligned}$$

where *N* is the *N*th-order interval. We make the assumption that the process $\gamma$ is aperiodic and the autocorrelation function ${{\,\mathrm{\mathbf {R}}\,}}\left( N\right) \rightarrow 0$ when $N \rightarrow \infty$, and so $\left( {{\,\mathrm{\mathbf {R}}\,}}_{N+1}-{{\,\mathrm{\mathbf {R}}\,}}_N\right) \rightarrow 0$. That is, the process decorrelates over long time-scales. Thus,

55 $$\begin{aligned} \lim _{N \rightarrow \infty } \sum _{k=1}^{N} z_k = {{\,\mathrm{\mathbf {R}}\,}}_0 - {{\,\mathrm{\mathbf {R}}\,}}_1\,. \end{aligned}$$

By summing Eq. (52) over all *k*, and inserting the result from Eq. (55), the limiting sum of the ISI serial correlation coefficients for the spike-train is

56 $$\begin{aligned} \sum _{k=1}^{\infty } \rho _k = -\frac{1}{2}\,. \end{aligned}$$
